# Clinical and cost‐effectiveness of eye movement desensitization and reprocessing for treatment and prevention of post‐traumatic stress disorder in adults: A systematic review and meta‐analysis

**DOI:** 10.1111/bjop.70005

**Published:** 2025-07-05

**Authors:** Emma Simpson, Christopher Carroll, Anthea Sutton, Jessica Forsyth, Annabel Rayner, Shijie Ren, Matthew Franklin, Emily Wood

**Affiliations:** ^1^ Sheffield Centre for Health and Related Research (SCHARR) University of Sheffield Sheffield UK

**Keywords:** eye movement desensitization and reprocessing, meta‐analysis, post‐traumatic stress disorder, systematic review

## Abstract

The objective was to provide up‐to‐date clinical and cost‐effectiveness evidence investigating eye movement desensitization and reprocessing (EMDR) for treatment or prevention of adult post‐traumatic stress disorder (PTSD). We conducted a systematic review of randomized controlled trials (RCTs) and cost‐effectiveness studies assessing PTSD symptoms in adults, published since the NICE 2018 guidelines. EMDR was compared with trauma‐focused‐cognitive behavioural therapy (TF‐CBT), waitlist or usual care. Six databases were searched in September 2023. Risk of bias was assessed. Data synthesis included Bayesian meta‐analyses of standardized mean differences if sufficient data were available from at least three RCTs. From 2038 records, 17 studies met the eligibility criteria. One modelling‐based study reported cost‐effectiveness, finding EMDR the most cost‐effective intervention compared to 10 others, including TF‐CBT. Sixteen RCTs (*n* = 1031) providing clinical PTSD outcome data were identified. Most studies had small sample sizes, and all but one was at high/moderate risk of bias. Additionally, 13 RCTs from NICE 2018 guidelines contributed to meta‐analyses. EMDR treatment was generally of shorter duration with a lower burden on patient time. Meta‐analyses found EMDR was statistically significantly better than waitlist/usual care. There was no significant difference in treatment effect between EMDR and TF‐CBT, both reported significantly improved PTSD symptoms.

## BACKGROUND

Eye movement desensitization and reprocessing (EMDR) is a psychological therapy that has been developed to resolve trauma (Shapiro, 2001). Currently, trauma‐focused cognitive behavioural therapy (TF‐CBT) is arguably the most common treatment approach for post‐traumatic stress disorder (PTSD). England's Talking Therapies services provided 30,441 finished courses of TF‐CBT for PTSD and 6675 finished courses of EMDR in 2022/23 (Shapiro, 2001). EMDR has been evaluated in adults with PTSD, commonly in single participant groups, such as first responders, (Morris et al., 2022) refugees or forcibly displaced people and (MacGowan et al., 2022) combatants (Kitchiner et al., 2019; Maglione et al., 2022). Other literature has focused on much broader PTSD populations as well as active and passive controls (Hoppen et al., 2023).

Systematic reviews and meta‐analyses have reported similar effectiveness for EMDR and TF‐CBT in treating PTSD (Hudays et al., 2022; Kitchiner et al., 2019; MacGowan et al., 2022; Maglione et al., 2022) or an immediate benefit for EMDR over TF‐CBT, which was no longer statistically significant at 3‐month follow‐up (Khan et al., 2018) There is less evidence for prevention of PTSD, (Skeffington et al., 2013) however, a review found EMDR was superior to no‐ or usual‐care or group debriefing and had similar effectiveness to trauma‐focused counselling (Bisson et al., 2013).

EMDR treatment has been found to be superior to some other trauma‐focused or non‐trauma‐focused psychological interventions and superior to waitlist (Hoppen et al., 2023; Hudays et al., 2022; Wilson et al., 2018). As a result, EMDR has been recommended as a therapy for PTSD in adults by several clinical bodies (American Psychiatric Association, 2010; National Institute for Health and Care Excellence, 2018; United States Department of Veterans Affairs & United States Department of Defense, 2017; World Health Organization, 2013). Both (International Society for Traumatic Stress Studies, 2018; United States Department of Veterans Affairs & United States Department of Defense, 2017) and European Society for Traumatic Stress Studies (ESTSS) (Roberts et al., 2023) recommend TF‐CBT, cognitive therapy or brief EMDR as first‐line treatment for PTSD. The UK National Institute for Health and Care Excellence (NICE) recommends TF‐CBT for PTSD, with EMDR offered for PTSD more than 3 months after a non‐combat‐related trauma or within 1–3 months of non‐combat‐related trauma where there is patient preference (National Institute for Health and Care Excellence, 2018).

The evidence base for EMDR for the prevention or treatment of PTSD is increasing constantly, meaning up‐to‐date systematic reviews are required if guidelines from clinical bodies are to remain valid and to determine whether new evidence contradicts or supports previous findings. NICE provide guidelines for health care in England and Wales, and a recent survey of psychiatrists found that their recommendations are widely used across Europe, notably by 37.8% of those surveyed from Western Europe, and more frequently than World Health Organization (WHO) or International Society for Traumatic Stress Studies (ISTSS) guidelines (with the exception of central Europe with approximately 25% frequency for both WHO and NICE guidelines) (Rojnic Kuzman et al., 2024). Cost‐effectiveness evidence also plays a critical role in NICE's decision‐making process, with the NICE review expressing that treatment choice for adults with PTSD had ‘potentially major resource implications’ (National Institute for Health and Care Excellence, 2018). Any more recent models or parameter updates will therefore be relevant to their recommendation.

As a result, the current systematic review and meta‐analysis was undertaken to identify the most up‐to‐date EMDR clinical and cost‐effectiveness evidence. The current systematic review seeks randomized controlled trials (RCT) (the gold standard for evaluating effectiveness) rather than including observational studies, is more recent (Hudays et al., 2022; Kitchiner et al., 2019; Maglione et al., 2022; McGowan et al., 2016; Morris et al., 2022; National Institute for Health and Care Excellence, 2018) and with a broader population than existing reviews (which have focussed on first‐responders, displaced people or military personnel only) (Kitchiner et al., 2019; MacGowan et al., 2022; Maglione et al., 2022; Morris et al., 2022) as well as with the inclusion of cost‐effectiveness, unlike other reviews (Rasines‐Laudes & Serrano‐Pintado, 2023).

The aim was to conduct a systematic review of evidence published since the 2018 NICE guidelines limited to RCT evidence for the effectiveness, safety and cost‐effectiveness of EMDR in the treatment or prevention of PTSD in adults, in comparison with alternative psychological treatments or no treatment. Secondary outcomes included discontinuations (a proxy for therapy acceptability), depression, anxiety, adverse events and quality of life.

## METHODS

The systematic review was undertaken in accordance with the general principles recommended in the York CRD guidance (Centre for Reviews and Dissemination, 2008) and the Preferred Reporting Items for Systematic Reviews and Meta‐Analyses (PRISMA) statement (Page et al., 2021). The review protocol is registered on the PROSPERO prospective register of systematic reviews as CRD42023463360. While restricted to EMDR studies, eligibility criteria mirrored those of the NICE 2018 guidelines (National Institute for Health and Care Excellence, 2018) although with the exception of excluding pharmacological comparators. NICE recommends psychological therapy as first‐line treatment, offering venlafaxine or a selective serotonin reuptake inhibitor where the service user has expressed a preference for drug treatment (National Institute for Health and Care Excellence, 2018). This paper presents the evidence for adults only; the evidence for children is reported elsewhere. For treatment, a diagnosis of PTSD was required; for prevention, the population had clinically significant PTSD symptoms following trauma, with sub‐threshold baseline scores on a validated scale (Table 1).

**TABLE 1:** Eligibility criteria.

Study design	For clinical effectiveness and safety, RCTs only; for cost‐effectiveness studies, the outcome is quality‐adjusted life years (QALYs)
Participants/population	Adults (age ≥18 years) with PTSD, either with diagnosis of PTSD according to Diagnostic and Statistical Manual of Mental Disorders (DSM), International Classification of Diseases (ICD) or similar criteria; or clinically significant PTSD symptoms as indicated by baseline scores above threshold on a validated scale more than 1 month after the traumatic event. For prevention studies, clinically significant PTSD symptoms following trauma, with subthreshold baseline scores on a validated scale
Intervention	Eye movement desensitization and reprocessing (EMDR)
Comparators	Any psychological trauma‐focused cognitive behavioural therapy (TF‐CBT), psychosocial therapy or non‐pharmacological therapy; waitlist; care as usual
Primary outcomes	PTSD symptoms/response/remission/relapse; Quality‐adjusted life years (QALYs)
Additional outcomes	Discontinuation for any reason (a proxy for acceptability of the intervention); Dissociative symptoms; Personal/social/occupational functioning (including global functioning/functional impairment); Sleeping difficulties; Quality of life; Symptoms of a coexisting condition (including anxiety, depression and substance misuse problems); Safety/adverse events (AEs); Treatment duration, patient time engaged with treatment
Publication date	2018 onwards (earlier RCT evidence was sourced from the comprehensive 2018 NICE evidence review for their guideline on PTSD (National Institute for Health and Care Excellence, 2018))
Exclusion	All other study designs. RCTs with fewer than *n* = 10 participants. Editorials, book chapters and conference papers and dissertations. Population with adjustment disorders; traumatic grief; psychosis as a coexisting condition; learning disabilities; PTSD during pregnancy or in the first year following childbirth; people in contact with the criminal justice system (not solely as a result of being a witness or victim) Studies of adolescents or children (A systematic review of the evidence in children and adolescents is the subject of a separate publication)

### Searches

Systematic searches were conducted in September 2023, on the following bibliographic databases: MEDLINE via Ovid, Embase via Ovid, PsycINFO via Ovid, Cochrane Library, CINAHL via EBSCO and PTSDpubs via ProQuest. The EMDR Publications Database maintained by the University of Sheffield for EMDR UK Members was also searched to cross‐check for any additional references not retrieved by searching the above listed bibliographic databases. Systematic searches were conducted to identify RCTs and cost‐effectiveness studies of EMDR for PTSD. A combination of subject headings and free‐text search terms relating to the population (adults with PTSD) and the intervention (EMDR) were combined with Boolean operators, and published methodological search filters were applied to identify RCTs, economic studies and systematic reviews. Searches were limited to 2018 onwards, to cover the evidence published since the NICE guidelines on PTSD (National Institute for Health and Care Excellence, 2018). Pre‐2018 RCTs were sourced from the comprehensive evidence underpinning the NICE guidelines (National Institute for Health and Care Excellence, 2018). The search was not limited by language, but non‐English language studies and abstracts were excluded at study selection unless they reported sufficient information for data extraction and quality assessment. The search strategy was developed on MEDLINE via Ovid, with input from clinical experts, then peer‐reviewed by a second information specialist using the PRESS checklist (McGowan et al., 2016). Searching also included reference list screening of included studies and relevant systematic reviews, and hand searching of key journals and websites. The full search strategies and sources can be found in Appendix S1. As this was intended as an adjunct to the quantitative findings of EMDR versus other psychological therapies (or no active therapy) reported in the 2018 NICE guidelines, (National Institute for Health and Care Excellence, 2018) the eligibility criteria applied had to be consistent with the NICE guidelines criteria.

#### Study selection, data extraction and risk of bias assessment

Identified records were imported into Covidence software (Covidence, n.d.). Study selection was conducted by two reviewers independently, at both title/abstract and full text stages using the eligibility criteria outlined in Table 1. These criteria were consistent with those applied in the production of the published NICE Clinical Guidelines (National Institute for Health and Care Excellence, 2018) to ensure that the present systematic review applied the same high standards as this report and could use the NICE evidence review as a robust source of relevant RCT evidence for the period up to 2018. Disagreements were resolved by consensus or reference to a subject expert if necessary. For clinical effectiveness and safety, data were extracted into a pre‐piloted data extraction form by one reviewer and checked by a second reviewer; disagreements were resolved by consensus or reference to a subject expert. The following data were tabulated: study characteristics, participant characteristics, intervention and comparator details and clinical outcome measures and results. Meta‐analyses were conducted for the primary outcome (PTSD) only. For these analyses, data were extracted from relevant identified RCTs, where available and in the appropriate format, and were combined with similar relevant data for the trials (up to 2018), extracted from the NICE evidence) (National Institute for Health and Care Excellence, 2018). The aim was to make use of all of the available data for the primary outcome and increase the power of the meta‐analyses.

For cost‐effectiveness studies, data were collected on study characteristics regarding publication (author, year, journal), study design (country, population, perspective [outcome and costs], analysis type [within‐trial and statistical methods used, or modelling/modelling‐type], outcome measure and associated detail [e.g. preference‐based measure, utility value set], time horizon, comparators, intervention duration, cost type, discount rates, year of valuation), study outcomes [results (QALYs/costs, incremental QALYs/incremental Costs, incremental cost‐effectiveness ratios (ICERs), probability of cost‐effectiveness) and sensitivity analysis].

For RCTs, quality assessment of the included studies was undertaken using the validated Cochrane Risk of Bias 2.0 tool (Higgins et al., 2024) for the primary outcome of our review. This quality assessment was conducted by one reviewer and checked by a second reviewer; disagreements were resolved by consensus or reference to a third reviewer if necessary.

#### Methods of data synthesis for clinical effectiveness

Where meta‐analysis was not possible, data were tabulated and reported in narrative synthesis. A minimum of three studies were required for statistical assessment via pairwise meta‐analysis (Dias et al., 2013). Studies could be from our review, or the review on which NICE guidance was based (National Institute for Health and Care Excellence, 2018). To be included in a pairwise meta‐analysis, a study had to include both mean and standard deviation (SD) for the change in PTSD from pre‐ to post‐treatment, or these data had to be calculable. The outcome considered was the change in PTSD symptoms before and after treatment and was expressed as a standardized mean difference (SMD) to enable comparison of PTSD symptoms using different scoring methods. Full details of the assumptions, calculations and statistical analyses conducted to assess treatment effect are provided in the Appendix S1. For the purposes of this review, positive change in SMD indicated improvement, and negative change in SMD indicated worsening of symptoms. All meta‐analyses were for adults with PTSD given delayed treatment (i.e. 3 months or more following trauma). There were no possible meta‐analyses for prevention or for early (within 3 months of event) treatment.

Since data were selected from studies from independent researchers, a common effect size could not be assumed and therefore a random effects model was used. Parameters of the random effects model were estimated using a Bayesian framework. Model and prior specification can be found in Appendix S1. All analyses were conducted using the freely available software WinBUGS (Lunn et al., 2000) via the R package, R2WinBUGS (Sturtz et al., 2005). Results are presented alongside the posterior median treatment effects and 95% credible intervals (CrI). Effect sizes were graded using Cohen's categories: not substantial (SMD < 0.2), small (0.2 ≤ SMD < 0.5), medium (0.5 ≤ SMD < 0.8), large (0.8 ≤ SMD) (Cohen, 2013). Study heterogeneity was graded and interpreted according to categories (Ren et al., 2018).

## RESULTS

### Search results

**FIGURE 1:**
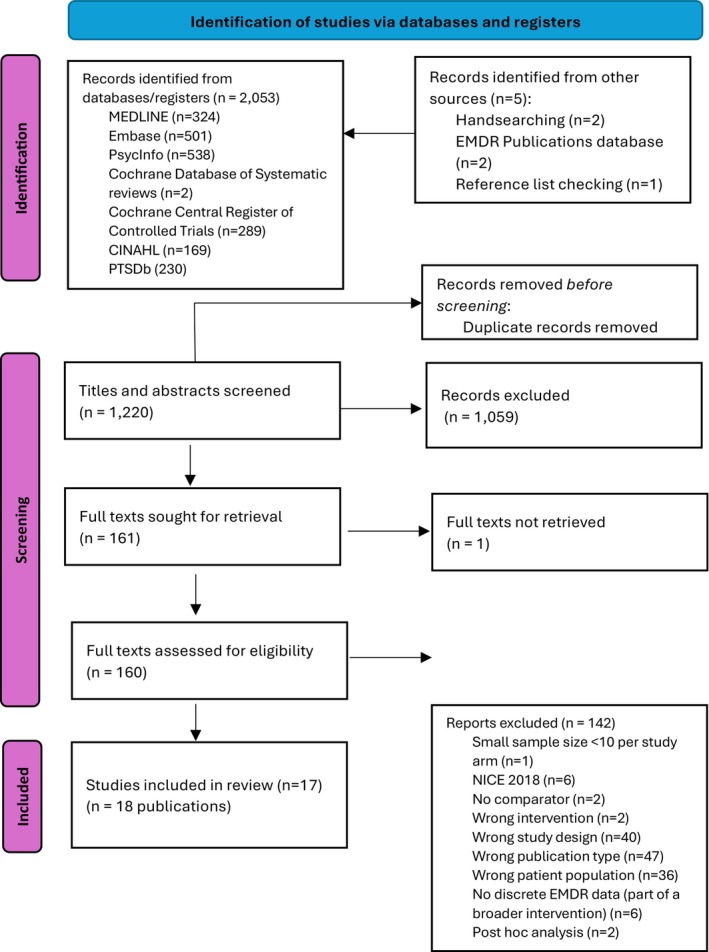
Flow diagram. From: Page et al. (2021).

Seventeen studies (with 18 publications) met the inclusion criteria for the review (Figure 1). Of these, one modelling‐based study reported cost‐effectiveness (Mavranezouli, Megnin‐Viggars, Grey, et al., 2020). The other 16 studies (17 publications) (Assmann et al., 2021; Bates et al., 2023; Boterhoven de Haan et al., 2020; Encinas et al., 2019; Farrell et al., 2023; Greenwald et al., 2021; Ironson et al., 2021; Jarero et al., 2018, 2019; Moghadam et al., 2020; Nijdam et al., 2018; Pérez et al., 2020; Rousseau et al., 2019; Santarnecchi et al., 2019; Shapiro et al., 2018; Stanbury et al., 2020; Zhao et al., 2023) reported clinical effectiveness data for PTSD outcomes (Figure 1).

### Clinical effectiveness results

Sixteen RCTs (with 17 publications) were identified by this systematic review to provide up‐to‐date data on clinical effectiveness of EMDR for PTSD outcomes in adults. Brief details of these trials are presented in Tables 2 and 3.

**TABLE 2:** Study characteristics of included trials from our search.

Study author, date, country	Intervention		Comparator 1		Comparator 2		Population early (within 3 months) or delayed treatment	Treatment or prevention	PTSD self‐report measure	PTSD clinician‐report measure	Follow‐up
	*n*	Details	*n*	Details	*n*	Details					
Bates et al. (2023) UK	13	EMDR (online): Up to 8 60–90 min sessions	13	CAU	NA	NA	Delayed	Prevention	PCL‐C	NR	6 months (post‐baseline)
Assmann et al. (2021), Boterhoven de Haan et al. (2020) International	67	EMDR: 12 × 90 min sessions, twice a week 6–8 weeks	72	ImRs: 12 × 90 min sessions, twice a week 6–8 weeks	NA	NA	Delayed	Treatment	IES‐R	CAPS‐5	Up to 1 year
Encinas et al. (2019) Mexico	14	EMDR‐PRECI: 6 × 1‐h sessions, twice daily during three consecutive days	12	CAU	NA	NA	Delayed	Treatment	PCL‐5	NR	90 days post‐treatment
Farrell et al. (2023) International	50	EMDR VGTEP: 4 × approx. 2 h sessions, intensive intervention over 1 week	45	WL (4 weeks)	NA	NA	Delayed	Treatment or prevention	ITQ	NR	6 months (post‐treatment)
Greenwald et al. (2021) USA	28	Intensive EMDR: several consecutive days, sometimes additional days	32	PC: As EMDR	NA	NA	Delayed	Treatment	TSI‐2	NR	12 weeks
Ironson et al. (2021) USA	34	EMDR: 4 90 min to 2 h sessions	37	Group‐administered SMT: 4 sessions; between 90 min and 2 h	34	SC (PFA): individual sessions between 90 min and 2 h	Early	Treatment	DTS	NR	6 months (post‐treatment)
Jarero et al. (2018) Mexico	35	EMDR‐IGPT‐OTS: 6× sessions during 2 consecutive days, 3× per day. first group session 1 h 40 min next sessions averaged 50 min	35	No treatment	NA	NA	Delayed	Treatment	PCL‐5	NR	90 days (post‐treatment)
Jarero et al. (2019) Mexico	30	EMDR‐PRECI: 2 × 1‐h treatment sessions on the same day	30	No treatment	NA	NA	Delayed	Treatment	PCL‐5	NR	90 days (post‐treatment)
Moghadam et al. (2020) Iran	15	EMDR: 4 sessions	15	CBT: 8 sessions	15	No active treatment	Delayed	Treatment	Mississippi PTSD measure	NR	Unclear
Nijdam et al. (2018) Netherlands	70	EMDR: Unspecified number of weekly sessions of 90 min	70	BEP: Up to 16 weekly sessions of 45 min	NA	NA	Delayed	Treatment	IES‐R	NR	17 weeks
Pérez et al. (2020) Mexico	40	EMDR‐IGTP (online). 4 × online group treatment sessions, every other day	40	WL	NA	NA	Early	Treatment	PCL‐C	NR	15 and 90 days after Waitlist treatment completion
Rousseau et al. (2019) France	18	EMDR. 1 × h session every 7–15 days	18	WL, supportive therapy 1 × h session every 7–15 days	NA	NA	Delayed	Treatment	PCLS	NR	Average 3 months (post‐baseline)
Santarnecchi et al. (2019) Italy	17	EMDR. average of 4 weeks (±2) of weekly sessions, 60 min	14	TF‐CBT: a skills‐based model; average of 10 weekly visits (±2)	NA	NA	Delayed	Treatment	DTS total score	CAPS	Unclear (‘post‐treatment’)
Shapiro et al. (2018) Israel	13	EMDR R‐TEP: 3 × 90 min sessions	12	WL	NA	NA	Early	Treatment	PCL‐5	NR	6 months (post‐treatment)
Stanbury et al. (2020) Australia	10	EMDR. 12 treatment sessions	10	PE. 12 sessions with homework	NA	NA	Delayed	Treatment	PCL‐C	CAPS	6 months
Zhao et al. (2023) China	28	EMDR: 12 weekly 90‐min sessions	29	WL	NA	NA	Delayed	Treatment	PCL‐C	CAPS	12 weeks (post‐treatment)

Abbreviations: BEP, brief eclectic psychotherapy; CAPS, Clinician Administered PTSD Scale; CAU, care as usual; CBT, cognitive behavioural therapy; DTS, Davidson Trauma Scale; EMDR, eye movement and desensitization reprocessing; IGTP‐OTS, Integrative Group Treatment Protocol‐Ongoing Traumatic Stress; ImRs, Imagery Rescripting; ITQ, International Trauma Questionnaire; NA, not applicable; NR, not reported; PC, progressive counting; PCL‐C, PTSD Checklist for DSM‐5 (PCL‐5) Civilian version; PE, prolonged exposure; PFA, Psychological First Aid; PRECI, Protocol for Recent Critical Incidents and Ongoing Traumatic Stress; PTSD, post‐traumatic stress disorder; RCT, randomized controlled trial; R‐TEP, recent traumatic episode protocol; SC, standard care; TF‐CBT, trauma‐focused CBT; TSI‐2, SMT: Stress Management with a Trauma focus; TSI‐2, Trauma Symptom Inventory 2; VGTEP, Video‐conference Group Traumatic Episode Protocol; WL, waitlist.

**TABLE 3:** Study characteristics of trials from NICE guidance included in meta‐analyses.

Study author, date, country	Intervention		Comparator 1		Population early (within 3 months) or delayed treatment	Treatment or prevention	PTSD measure
	*n*	Details	*n*	Details			
Acarturk et al. (2015)	15	EMDR	14	WL	Delayed	Treatment	Self‐report
Acarturk et al. (2016)	49	EMDR	49	WL	Delayed	Treatment	Self‐report
Aldahadha et al. (2012)	25	EMDR	26	WL	Delayed	Treatment	Self‐report
Capezzani et al. (2013)	11	EMDR	10	TF‐CBT	Delayed	Treatment	Self‐report and clinician‐report
Carlson et al. (1998)	10	EMDR	12	WL	Delayed	Treatment	Self‐report
Edmond and Rubin (2004), Edmond et al. (1999)	20	EMDR	19	WL	Delayed	Treatment	Self‐report
Himmerich et al. (2016)	21	EMDR	17	WL	Delayed	Treatment	Self‐report
Laugharne et al. (2016)	10	EMDR	10	TF‐CBT	Delayed	Treatment	Clinician‐report
Nijdam et al. (2012)	51	EMDR	42	TF‐CBT	Delayed	Treatment	Clinician‐report
Power et al. (2002)	27	EMDR	21 24	TF‐CBT WL	Delayed	Treatment	Self‐report
Rothbaum et al. (2005)	20	EMDR	20 20	TF‐CBT WL	Delayed	Treatment	Self‐report and clinician‐report
Taylor et al. (2003)	15	EMDR	15	TF‐CBT	Delayed	Treatment	Self‐report and clinician‐report
Yurtsever et al. (2018)	18	EMDR	29	WL	Delayed	Treatment	Self‐report

Most studies had two treatment arms; however, two studies included both TF‐CBT and waitlist/usual care comparators (Ironson et al., 2021; Moghadam et al., 2020). All active comparators were CBT‐based. All studies were open‐label; most were single‐centre studies, and four were multi‐centre (Boterhoven de Haan et al., 2020; Pérez et al., 2020; Rousseau et al., 2019; Stanbury et al., 2020). There were two international trials, (Boterhoven de Haan et al., 2020; Farrell et al., 2023) other trials were from Mexico, (Encinas et al., 2019; Jarero et al., 2018, 2019; Pérez et al., 2020) the USA, (Greenwald et al., 2021; Ironson et al., 2021) Australia, (Stanbury et al., 2020) China, (Zhao et al., 2023) France, (Rousseau et al., 2019) Iran, (Moghadam et al., 2020) Israel, (Shapiro et al., 2018) Italy, (Santarnecchi et al., 2019) the Netherlands and the UK (Bates et al., 2023). Follow‐up ranged from post‐treatment to 1 year, with most trials having 3‐ or 6‐month follow‐up.

Duration of therapy between arms was either similar or of shorter duration for EMDR. Stanbury reported that for therapy and homework hours, there was less average time spent in therapy for EMDR, 20.65 h (SD = 3.07), than for prolonged exposure, 63.20 h (SD = 23.97), with similar effectiveness between treatment arms (Stanbury et al., 2020). Santarnecchi reported dose response, with treatment arms reporting similar effectiveness, with fewer than half the number of sessions needed for EMDR (4 weekly sessions ±2) compared to TF‐CBT (10 weekly sessions ±2) (Santarnecchi et al., 2019). Moghadam 2020 provided half the number of sessions for EMDR (4 sessions) than for TF‐CBT (8 sessions) (Moghadam et al., 2020).

Participant characteristics are detailed in Table S1. Populations included the following: victims of criminal injury, violence and assault, including sexual and domestic violence; (Boterhoven de Haan et al., 2020; Greenwald et al., 2021; Ironson et al., 2021; Nijdam et al., 2018; Rousseau et al., 2019; Stanbury et al., 2020; Zhao et al., 2023) combat; (Moghadam et al., 2020) natural disasters or war; (Nijdam et al., 2018; Santarnecchi et al., 2019; Stanbury et al., 2020) parents of children with chronic conditions; (Encinas et al., 2019) adults with a cancer diagnosis (Jarero et al., 2018); first responders or equivalent, including front‐line health professionals during the COVID‐19 pandemic (Farrell et al., 2023) and COVID‐19 hospitalization (Bates et al., 2023). Where reported, the trauma concerned a single event in three studies; (Ironson et al., 2021; Nijdam et al., 2018; Santarnecchi et al., 2019) multiple events in five studies (Greenwald et al., 2021; Moghadam et al., 2020; Pérez et al., 2020; Shapiro et al., 2018; Zhao et al., 2023) and participants with single and multiple prior traumatic events in one study (Boterhoven de Haan et al., 2020).

For the purposes of rendering the comparisons more homogenous and robust, and in accordance with the previous NICE guidelines, (National Institute for Health and Care Excellence, 2018) the studies were grouped and analysed according to the following criteria: whether the comparator was a form of TF‐CBT or waitlist/usual care/no treatment; whether treatment was delayed (more than 3 months after traumatic event) or early (within 3 months of event); follow‐up post‐treatment or later duration at data collection and whether the PTSD outcome scale was self‐report or clinician‐assessed (Tables S2–S8).

To be included in a pairwise meta‐analysis, a study had to include both mean and standard deviation (SD) for the change in PTSD from pre‐ to post‐treatment, or these data had to be calculable. Some of the studies were therefore not eligible for inclusion in meta‐analyses. A minimum of three studies were required for statistical assessment via pairwise meta‐analysis. Fourteen references for 13 trials were also included in these meta‐analyses from the previously published NICE guidelines. This systematic review therefore includes a total of 29 relevant clinical RCTs evaluating EMDR in adults with PTSD.

### Risk of bias assessments

**FIGURE 2:**
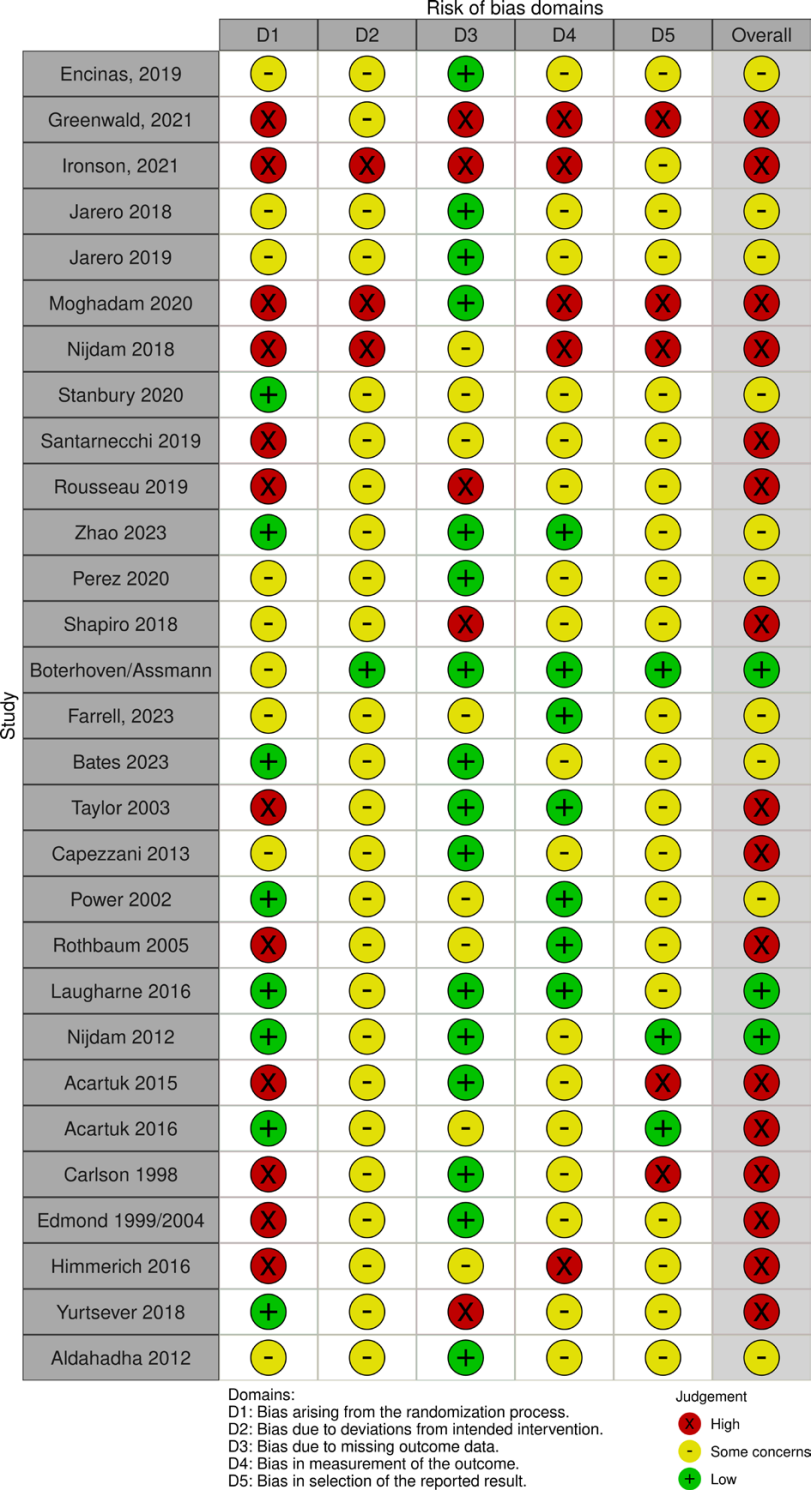
Cochrane risk of bias assessment of included studies.

All studies were open‐label; most were single‐centre studies. Overall, nine included studies were at moderate risk of bias (Bates et al., 2023; Encinas et al., 2019; Farrell et al., 2023; Jarero et al., 2018, 2019; Pérez et al., 2020; Shapiro et al., 2018; Stanbury et al., 2020; Zhao et al., 2023). Six studies were at high risk of bias (Greenwald et al., 2021; Ironson et al., 2021; Moghadam et al., 2020; Nijdam et al., 2018; Rousseau et al., 2019; Santarnecchi et al., 2019). Only one of the studies was at low risk of bias (Boterhoven de Haan et al., 2020). Poor reporting was the driver of these assessments. The principal source of bias within the trials related to the randomisation process, which was rarely well reported or used independent approaches to randomisation or allocation concealment. The baseline details of participants in different arms were also sometimes not reported, so potential imbalances in prognostic factors could not be judged. The frequent absence of trial protocols also raised some concerns about potential selective reporting. Outcome measures were generally well known, validated and robust, although trials were unblinded and outcomes were often self‐reported. Missing data rarely presented a problem due to the short duration of treatment and follow‐ups (commonly 3–6 months). The same process was conducted on RCTs identified from the NICE evidence review that were included in the current systematic review. For a full risk of bias summary, see Figure 2 (created in robvis software) (McGuinness & Higgins, 2020). There was no clear correlation between risk of bias and direction of effect of PTSD results.

### Results of PTSD symptoms

The PTSD results for all 16 included studies were tabulated (Tables S2–S8). In total 1031 patients contributed PTSD data, of which *n* = 496 were randomly assigned to EMDR, *n* = 252 to TF‐CBT, and *n* = 283 were assigned to waitlist/usual care. There was heterogeneity in populations and comparators for all the comparisons. From our review, six studies were eligible to be included in pairwise meta‐analyses (Boterhoven de Haan et al., 2020; Encinas et al., 2019; Jarero et al., 2018, 2019; Rousseau et al., 2019; Stanbury et al., 2020).

#### EMDR versus TF‐CBT as delayed treatment (more than 3 months after traumatic event), PTSD self‐report measures, post‐treatment

Seven studies provided data for this comparison (Table S2) (Boterhoven de Haan et al., 2020; Greenwald et al., 2021; Ironson et al., 2021; Moghadam et al., 2020; Nijdam et al., 2018; Santarnecchi et al., 2019; Stanbury et al., 2020). There was heterogeneity between studies in the type of TF‐CBT and PTSD self‐report scale used. Across studies, both EMDR and TF‐CBT groups improved after treatment. For the comparison between groups, significance values were not widely reported. Moghadam et al. reported significantly more improvement for EMDR over TF‐CBT. For the remaining studies, where reported, there was no significant time and treatment group interaction, indicating similar levels of improvement for both groups. The study with a low risk of bias (Boterhoven de Haan et al., 2020) found similar improvements for EMDR and imagery rescripting post‐treatment, and both treatment arms retained the improvement at 1‐year follow‐up. Duration of therapy between arms was either similar or of shorter duration for EMDR. Stanbury et al. (2020) reported that for therapy and homework hours, there was less average time spent in therapy for EMDR, 20.65 h (SD = 3.07), than for prolonged exposure, 63.20 h (SD = 23.97), with similar effectiveness between treatment arms. Santarnecchi et al. (2019) reported dose response, with treatment arms reporting similar effectiveness, with fewer than half the number of sessions needed for EMDR (4 weekly sessions ±2) compared to TF‐CBT (10 weekly sessions ±2). Moghadam et al. (2020) provided half the number of sessions for EMDR than for TF‐CBT.

**FIGURE 3:**
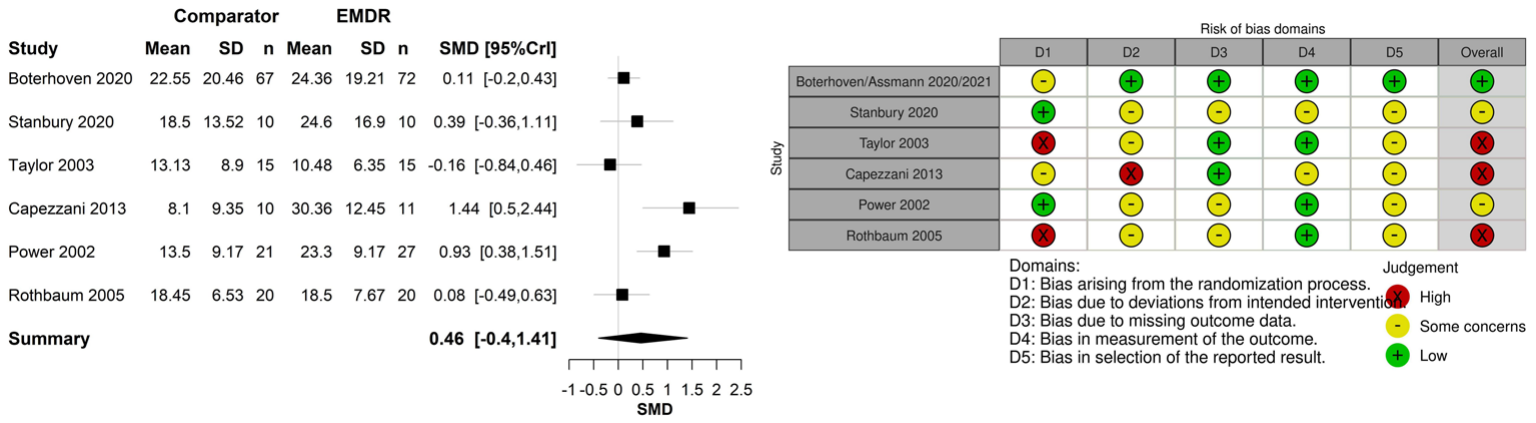
Standardized mean difference for EMDR relative to TF‐CBT using self‐reported scores post‐treatment.

Two of these studies were eligible for inclusion in meta‐analyses, (Boterhoven de Haan et al., 2020; Stanbury et al., 2020) and with the relevant trials from the NICE evidence, (Capezzani et al., 2013; Power et al., 2002; Rothbaum et al., 2005; Taylor et al., 2003) there were enough trials to make meta‐analysis viable. Data were from 298 patients. One trial had a low risk of bias; the others were at moderate risk of bias. Figure 3 presents the SMD of EMDR relative to TF‐CBT using self‐reported scores. The population effect of EMDR was SMD 0.46 (95% CrI −0.40 to 1.41). The result suggests a potential beneficial effect with a small effect size of EMDR compared to TF‐CBT (self‐reported scores); however, the result was not statistically significant. The between‐study standard deviation was estimated to be 0.81 (95% CrI 0.29–2.11), which implies an extremely high heterogeneity between studies, such that the treatment effect in one study is at least 50 times that of another study.

#### EMDR versus TF‐CBT as delayed treatment, PTSD clinician‐report measures, post‐treatment

Three studies reported data for this comparison (Table S3) (Boterhoven de Haan et al., 2020; Santarnecchi et al., 2019; Stanbury et al., 2020). Across studies, both EMDR and TF‐CBT groups improved after treatment. There was heterogeneity between studies in type of TF‐CBT. There was no significant time and treatment group interaction for any of the studies, indicating similar levels of improvement for both groups. Two studies (Santarnecchi et al., 2019; Stanbury et al., 2020) both had a shorter duration of treatment for EMDR than for TF‐CBT.

**FIGURE 4:**
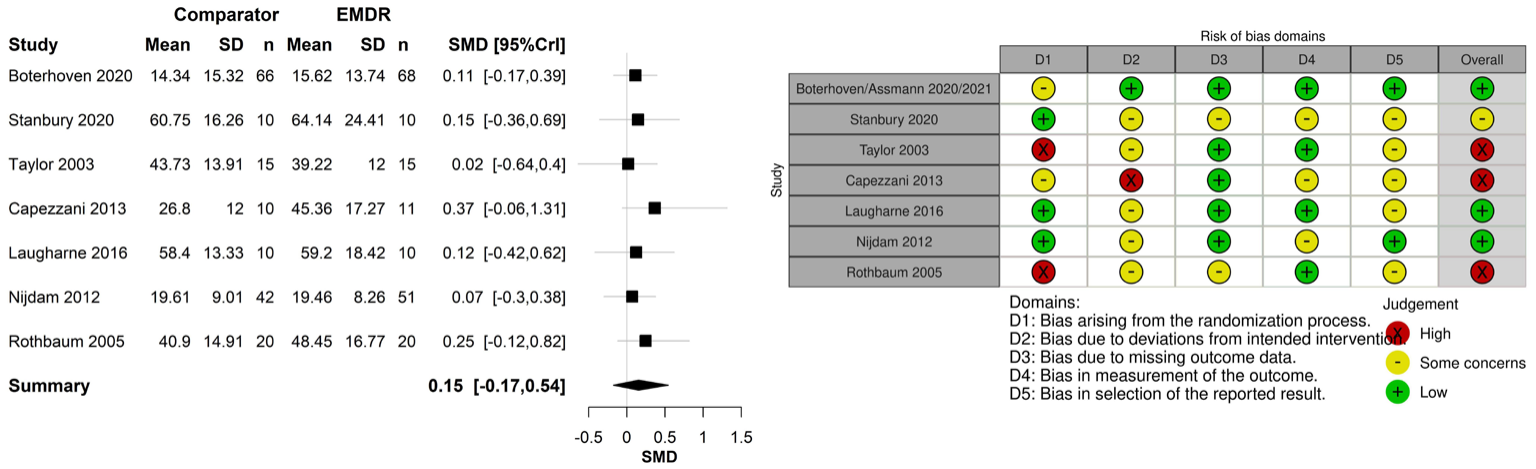
Standardized mean difference for EMDR relative to TF‐CBT using self‐reported scores post‐treatment.

Two of these studies were eligible for inclusion in meta‐analyses (Boterhoven de Haan et al., 2020; Stanbury et al., 2020) and with the relevant trials from the NICE evidence (Capezzani et al., 2013; Laugharne et al., 2016; Nijdam et al., 2012; Rothbaum et al., 2005; Taylor et al., 2003) there were enough trials to make meta‐analysis viable. Data were from 358 patients. Three of these studies were at low risk of bias, (Boterhoven de Haan et al., 2020; Laugharne et al., 2016; Nijdam et al., 2012) the others at moderate risk of bias. Figure 4 presents the SMD of EMDR relative to TF‐CBT using clinician rated scores. The population effect of EMDR was SMD 0.15 (95% CrI −0.17 to 0.54). The result suggests a potential beneficial effect with a non‐substantial effect size of EMDR compared to TF‐CBT when using clinician rated scores; however, the result was not statistically significant. The between‐study standard deviation was estimated to be 0.24 (95% CrI 0.01–0.92), which implies moderate heterogeneity between studies, such that the treatment effect in one study could be 1.48–7.10 times that of another study.

#### EMDR versus TF‐CBT as early treatment, PTSD clinician‐report measures, post‐treatment

One study (Ironson et al., 2021) reported data on early (within 3 months of event) treatment, using a PTSD self‐report scale. This study had three arms, EMDR, TF‐CBT and treatment as usual (Tables S4 and S7). There was improvement in the EMDR and TF‐CBT groups post‐treatment with a lower level of improvement in the treatment as usual group, however the improvement in the ‘treatment as usual group’ at 6‐month follow‐up was sufficient to mean there was no significant treatment group by time interaction. Treatment duration was similar for EMDR and TF‐CBT. The study did not provide data eligible for meta‐analysis (SD was not reported).

#### EMDR versus waitlist/usual care as delayed treatment, PTSD self‐report measures, post‐treatment

Seven studies provided data for this comparison (Table S5) (Encinas et al., 2019; Farrell et al., 2023; Jarero et al., 2018, 2019; Moghadam et al., 2020; Rousseau et al., 2019; Zhao et al., 2023). There was some heterogeneity in the PTSD self‐report scale used, although four studies used PCL‐5. In all studies, the EMDR group improved after treatment. In one study (Encinas et al., 2019), the usual care group deteriorated during the study period. There was significantly more improvement in the EMDR group than in the comparator group for all seven studies that reported significance levels.

Four of these studies provided eligible data for meta‐analysis (Encinas et al., 2019; Jarero et al., 2018, 2019; Rousseau et al., 2019) for the comparison pre‐ to post‐treatment. Nine relevant studies from the NICE evidence were added (Acarturk et al., 2015, 2016; Aldahadha et al., 2012; Carlson et al., 1998; Edmond et al., 1999; Edmond & Rubin, 2004; Power et al., 2002; Rothbaum et al., 2005; Yurtsever et al., 2018). One study from the NICE evidence (Jensen, 1994) was excluded due to not reporting a standardized mean difference (see Table S5). Data were from 586 patients. Seven of these studies were at high risk of bias, (Acarturk et al., 2015, 2016; Carlson et al., 1998; Edmond et al., 1999; Edmond & Rubin, 2004; Himmerich et al., 2016; Rousseau et al., 2019; Yurtsever et al., 2018), and the others were at moderate risk of bias.

**FIGURE 5:**
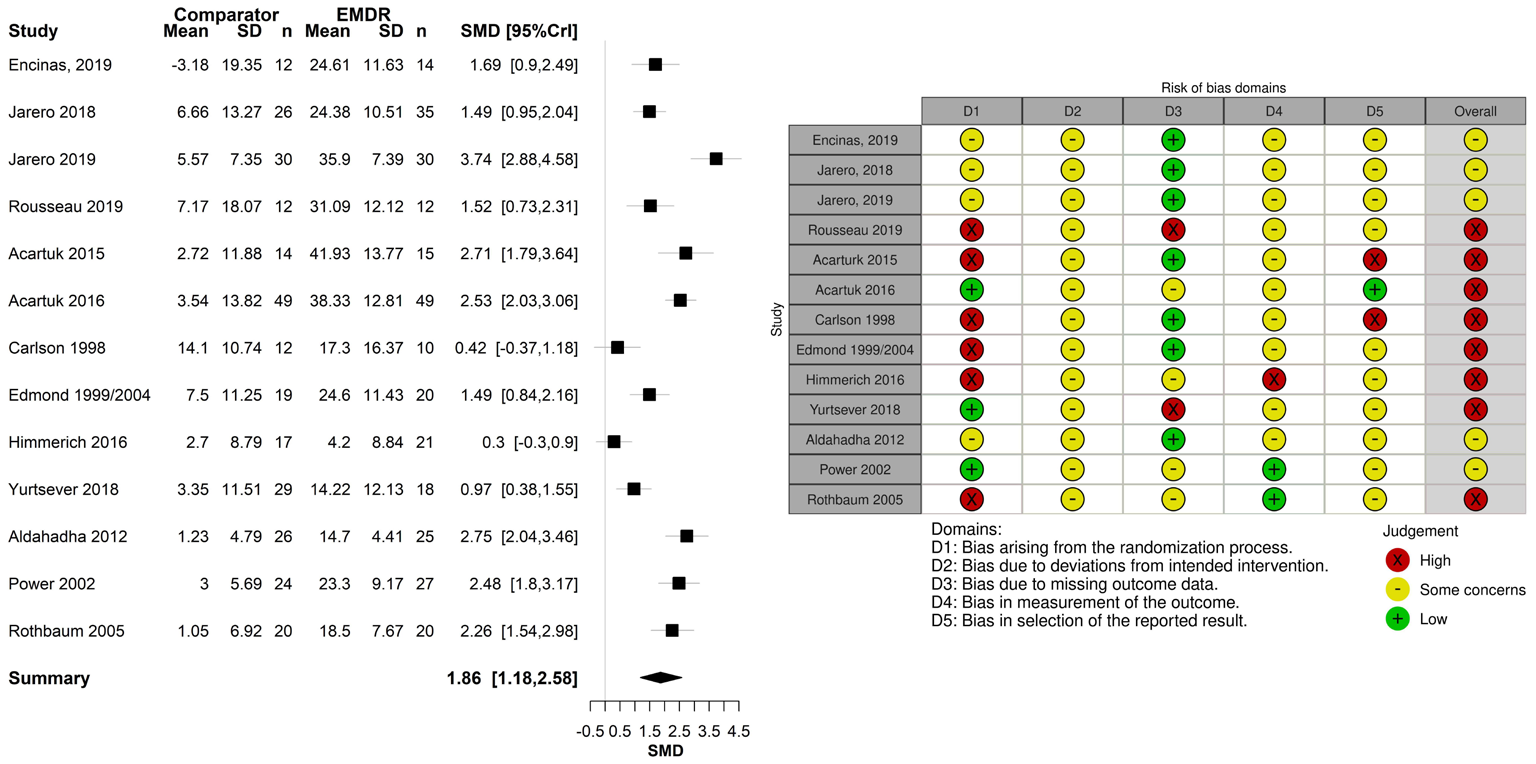
Standardized mean difference for EMDR relative to waitlist/usual care using self‐reported scores post‐treatment.

Figure 5 presents the SMD of EMDR relative to waitlist/usual care using self‐reported scores. The population effect of EMDR was SMD 1.86 (95% CrI 1.18–2.58). The result suggests a statistically significant beneficial effect with a large effect size of EMDR compared to waitlist/usual care. The between‐study standard deviation was estimated to be 1.13 (95% CrI 0.72–1.92), which implies extremely high heterogeneity between studies, such that the treatment effect in one study is at least 50 times that of another study.

#### EMDR versus waitlist/usual care as delayed treatment, PTSD self‐report measures, 3‐month follow‐up

For this comparison, with 3‐month follow‐up, three studies provided data for meta‐analysis (Encinas et al., 2019; Jarero et al., 2018, 2019). Moghadam et al. (2020) was not included in the meta‐analysis due to unclear reporting; it was unclear which one of the subscales of the Mississippi PTSD measure was used. Zhao et al. (2023) was not included in the meta‐analysis as the population was at high risk of psychosis, and therefore, differed from populations in the other studies included within the meta‐analysis. Data were from 147 patients.

**FIGURE 6:**
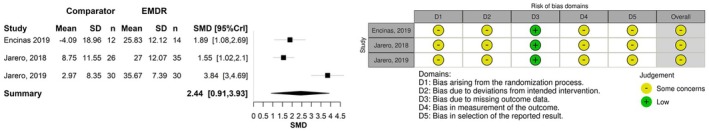
Standardized mean difference for EMDR relative to waitlist/usual care at 3‐month follow‐up using self‐reported scores.

Due to the low study number, an alternative, more informative, log‐normal prior was used for the population treatment effect standard deviation (τ) (see Appendix S1). A pairwise meta‐analysis was performed to evaluate the overall population effect of EMDR compared to waitlist/usual care at a follow‐up time of 3 months. Figure 6 presents the SMD of EMDR relative to waitlist/usual care at a follow‐up time of 3 months using self‐reported scores. The population effect of EMDR was SMD 2.44 (95% CrI 0.91–3.93). The result suggests a statistically significant beneficial effect with a large effect size of EMDR compared to waitlist/usual care at a follow‐up time of 3 months. The between‐study standard deviation was estimated to be 1.18 (95% CrI 0.77–1.82), which implies extremely high heterogeneity between studies, such that the treatment effect in one study is at least 50 times that of another study.

#### Treatment, waitlist/usual care, delayed, PTSD clinician report

No meta‐analyses were possible for this comparison. One study (Zhao et al., 2023) reported data on this comparison and reported a significant advantage for EMDR over waitlist (Table S6). It was not eligible for meta‐analysis as the population were at high risk of psychosis (although without a current or past diagnosis of a psychotic disorder) and therefore differed from other studies in the review.

#### Treatment, waitlist/usual care, early (within 3 months of event), PTSD self‐report

No meta‐analyses were possible for this comparison. Three studies provided data for this comparison (Table S7) (Ironson et al., 2021; Pérez et al., 2020; Shapiro et al., 2018). In all three studies, the EMDR group improved after treatment. In two studies (Pérez et al., 2020; Shapiro et al., 2018), the waitlist group deteriorated during the pre‐ to post‐treatment period. There was significantly more improvement in the EMDR group than the comparator group for one study (Pérez et al., 2020). A third study (Ironson et al., 2021) reported that on the Post Traumatic Cognitions Inventory (PTCI), EMDR was significantly better than usual care at 1‐ and 3‐month follow‐up, but not at 6‐month follow‐up (group × time interaction *p* = .039). Not all trials provided standard deviation data, and so meta‐analysis was precluded.

#### Prevention (sub‐threshold PTSD), waitlist/usual care, delayed, PTSD self‐report

No meta‐analyses were possible for this comparison. Only one study provided data for this comparison (Bates et al., 2023) (Table S8), and so meta‐analysis was not possible. There was improvement in the EMDR arm, and deterioration in the usual care arm, at 6‐month follow‐up.

The findings regarding other clinical outcomes explored by this review (discontinuation rates, adverse events, depression, anxiety, functioning, health‐related quality of life) are reported in the Appendix S1 (Tables S9–S12).

The evidence suggests EMDR has a low discontinuation rate which is generally comparable to or slightly better than comparator therapies. Adverse events were very rare. EMDR significantly improved depression more than waitlist or usual care, and functioning or health‐related quality of life were rarely reported.

#### Cost‐effectiveness

None of the RCTs included in the clinical effectiveness section provided a within‐trial cost‐effectiveness analysis, which would have given insight into the cost of delivering the intervention, alongside the causal impact of future costs and general health‐related outcomes. One modelling‐based cost‐effectiveness study was identified by the search (Mavranezouli, Megnin‐Viggars, Daly, et al., 2020). This was based on a network meta‐analysis that included 71 RCTs for changes in PTSD symptom scores between baseline and treatment endpoint, and 28 RCTs for changes in PTSD symptom scores between baseline and 1–4‐month follow‐up (Hoppen et al., 2023).

The data extraction for this study is available in Table S13. The model used a hybrid decision‐analytic model of a decision tree followed by a Markov model. The cohort had a starting age of 39, with 51.6% women. The population concerned those presenting with clinically important PTSD with symptoms present for more than 3 months after the incidence (as for the delayed treatment comparisons of the clinical evidence).

EMDR was the most cost‐effective intervention in the adult population compared to 10 other interventions, including TF‐CBT and no treatment (Table S14). The deterministic sensitivity analysis showed that when using alternative values for risk of relapse, utility and costs, the results remained robust. In the probabilistic sensitivity analysis, EMDR remained the most cost‐effective option.

## DISCUSSION

There was a beneficial effect for both EMDR and TF‐CBT on PTSD symptoms post‐treatment. Results of meta‐analyses found that, for treatment more than 3 months following a traumatic event, there was only a small, non‐statistically significant potential beneficial effect for EMDR over TF‐CBT. This was the case both for studies using self‐report measures of PTSD, SMD 0.46 (95% CrI −0.40 to 1.41), and for studies using clinician‐rated measures of PTSD, SMD 0.15 (95% CrI −0.17 to 0.54). Similar effectiveness was seen despite EMDR requiring either a shorter duration of therapy than TF‐CBT or a similar number of sessions.

Results of meta‐analyses found, for treatment more than 3 months following event, EMDR was statistically significantly better than waitlist/usual care in reducing PTSD symptoms, as measured with self‐report scales. This benefit was seen post‐treatment, SMD 1.86 (95% CrI 1.18–2.58) and at 3‐month follow‐up, SMD 2.44 (95% CrI 0.91–3.93).

While EMDR and TF‐CBT were similarly effective in reducing symptoms of PTSD, EMDR often had a shorter duration of therapy than TF‐CBT. Having fewer sessions reduces the cost to providers in terms of therapist time. EMDR also has a lower burden on patient time, as no homework is required after sessions. It is also easier to be assured of treatment fidelity for treatments where unsupervised homework is not required. EMDR was significantly better in reducing symptoms of PTSD than waitlist/usual care, with effect immediately post‐treatment and at later follow‐up. EMDR was the most cost‐effective intervention, compared to 10 other interventions, including TF‐CBT and no treatment, based on a modelling‐based study that was driven by network meta‐analysis effectiveness evidence and other synthesized evidence. There was a very low rate of treatment discontinuation for EMDR, adverse events were rare, and EMDR demonstrated benefits for depression and anxiety.

This current review includes more RCTs (29 clinical RCTs) than any previous review of EMDR and, unlike other reviews, has a broader population and contains a cost‐effectiveness review (Hoppen et al., 2023; Hudays et al., 2022; Kitchiner et al., 2019; MacGowan et al., 2022; Maglione et al., 2022; Morris et al., 2022; Rasines‐Laudes & Serrano‐Pintado, 2023). Some previous EMDR reviews had narrower populations and fewer RCTs: Morris et al. (2022), MacGowan et al. (2022) and Hudays et al. (2022) had 3–5 RCTs in adults; Wright et al. (2024) found 15 RCTs, of which 8 were available for analysis; Rasines‐Laudes and Serrano‐Pintado (2023) included pharmacology as well as psychological therapy comparators and found 18 RCTs. Therefore, this paper currently represents an up‐to‐date, comprehensive and rigorous systematic review for EMDR as a therapy for adults with PTSD.

Overall, results were similar to those in the 2018 NICE review (National Institute for Health and Care Excellence, 2018). One additional meta‐analysis was feasible in our review for the comparison treatment, waitlist/usual care, delayed, PTSD self‐report, follow‐up 3 months following treatment. This found a significant benefit of EMDR over waitlist/usual care. The only cost‐effectiveness study identified as part of this review was the published version of the model used in the NICE review. Although the model structure was the same, some of the parameter inputs on clinical effectiveness and the choice of comparator interventions differed, meaning that the NICE model initially suggested that TF‐CBT individual <8 sessions was the most cost‐effective. By contrast, the published version of the model included in our review found that EMDR was the most cost‐effective intervention.

To be consistent with an existing review, specific inclusion/exclusion criteria were applied, meaning some RCTs of EMDR have been omitted. For example, comorbid psychosis was excluded, meaning clinical data from van den Berg et al. (2015) (which found EMDR and prolonged exposure were equally effective) and cost data from de Bont et al. (2019) and van den Berg et al. (2016) were excluded. de Bont et al. (2019) and van den Berg et al. (2016) found EMDR and prolonged exposure had similar clinical effectiveness, but EMDR had lower costs; findings somewhat aligned with those of our review, in which EMDR was found to be the most clinically effective option while also being one of the cheapest. The evidence review eligibility criteria of the ISTSS guidelines (International Society for Traumatic Stress Studies, 2018) differed from those of the NICE review, for example, not restricting by sample size. However, both ISTSS and NICE recommend EMDR and TF‐CBT for the treatment of PTSD in adults (International Society for Traumatic Stress Studies, 2018; National Institute for Health and Care Excellence, 2018). Overall, the evidence base is remarkably consistent, regardless of differences in inclusion criteria. A recent review by Hoppen et al. (2023) also found EMDR was better than waitlist and had similar effectiveness to TF‐CBT, even though this focused on a specific comparison only (participants with single vs. multiple trauma). Also, a recent review by Wright et al. (2024) of a smaller sample of eight studies with individual patient data only found EMDR to have a similar treatment effect to other psychological therapies.

### Limitations

This review was not a complete update of the NICE guidelines, being restricted to adults and excluding pharmacology. In terms of the analyses, we limited the inclusion of older RCTs to those enabling meta‐analysis of data for the primary outcome only. In terms of the evidence overall, most of the identified RCTs had small sample sizes and were judged to be at high or moderate risk of bias, with only one RCT having a low risk of bias. There are still fewer RCTs of EMDR than for CBT in terms of what has been identified by previous reviews (Hoppen et al., 2023; National Institute for Health and Care Excellence, 2018). There was heterogeneity in patient groups, comparators and outcome measures used in trials. Most trials were conducted outside Europe, meaning there is a possibility that populations differ from the target population in Europe. This review was limited to English language publications, and previously published trials (pre 2018) were sourced from a prior high‐quality systematic review. While accepting that one study focused exclusively on females, (Jarero et al., 2018) generally there was little or no analysis of personal or socioeconomic characteristics that shape or determine health opportunities or outcomes (O'Neill et al., 2014). However, this systematic review was conducted by applying the highest international standards, applying strict, policy‐relevant criteria (consistent with UK NICE guidelines), and identified and analysed more up‐to‐date RCT evidence from a broader population base than previously published systematic reviews.

### Implications for policy and practice

The available evidence, while there being fewer trials for EMDR relative to CBT, does indicate that, in the treatment of PTSD in adults, EMDR demonstrates comparable effectiveness to TF‐CBT, which is the current first choice therapy according to guidelines, such as those from NICE. This is demonstrated by direct comparison in RCTs of EMDR compared to TF‐CBT, with evidence synthesis for delayed treatment. Further research is needed for early treatment with EMDR. Furthermore, the current research suggests the potential for EMDR to be the more a cost‐effective intervention for PTSD in adults, which, if substantiated by further robust evidence, could have implications for treatment duration, service delivery and ultimately, patient outcomes. However, these potential implications require further follow‐up studies to inform definitive recommendations for policy and widespread clinical practice.

## CONCLUSION

This systematic review identified and analysed evidence from 29 RCTs in total comparing EMDR with either other therapies or no active treatment in adults with PTSD. This systematic review also identified one cost‐effectiveness study that found EMDR to be a cost‐effective treatment option. The review found that EMDR significantly reduced PTSD symptoms, equivalent to the accepted standard‐of‐care, TF‐CBT, and that it was superior to all alternative therapies evaluated. EMDR also had low discontinuation rates and had a lower time burden for patients than TF‐CBT.

## AUTHOR CONTRIBUTIONS

**Emma Simpson:** Conceptualization; methodology; data curation; investigation; formal analysis; funding acquisition; project administration; writing – original draft. **Christopher Carroll:** Conceptualization; methodology; data curation; investigation; formal analysis; funding acquisition; writing – review and editing. **Anthea Sutton:** Conceptualization; methodology; data curation; investigation; formal analysis; funding acquisition; project administration; writing – review and editing. **Jessica Forsyth:** Methodology; data curation; investigation; formal analysis; writing – review and editing. **Annabel Rayner:** Methodology; data curation; investigation; formal analysis; writing – review and editing. **Shijie Ren:** Conceptualization; methodology; supervision; funding acquisition; writing – review and editing. **Matthew Franklin:** Conceptualization; methodology; investigation; formal analysis; supervision; funding acquisition; writing – review and editing. **Emily Wood:** Conceptualization; supervision; funding acquisition; writing – review and editing.

## Supporting information


Appendix S1

## Data Availability

All data extracted are included in the manuscript and supporting information (Appendix S1).
