# Supplementary material for: Clinical and cost‐effectiveness of eye movement desensitization and reprocessing for treatment and prevention of post‐traumatic stress disorder in adults: A systematic review and meta‐analysis
Source: Br J Psychol. 2025 Jul 5;116(4):1128–49. doi: 10.1111/bjop.70005 (PMC12514334; doi:10.1111/bjop.70005)
Supplement: Supplementary file 1 — Appendix S1 [file BJOP-116-1128-s001.docx]

**Supporting information**

- **Search Strategies**
- **Methods of data synthesis for clinical effectiveness**
- **Participant characteristics of included studies (Table S1)**
- **PTSD results tables (Tables S2-S8)**
- **Discontinuations (Table S9)**
- **Adverse events (Table S10)**
- **Depression and anxiety (Table S11)**
- **Health-related Quality of Life and Functioning (Table S12)**
- **Cost effectiveness (Tables S13, S14)**

**Search Strategies**

**MEDLINE**

Ovid MEDLINE(R) and Epub Ahead of Print, In-Process, In-Data-Review & Other Non-Indexed Citations and Daily <1946 to September 14, 2023>

1 exp Stress Disorders, Post-Traumatic/ 41583

2 PTSD.ti,ab. 32537

3 moral* injur*.ti,ab. 676

4 exp Combat Disorders/ 3219

5 ((combat or battle or conflict or war or wars) adj5 (stress or disorder* or neuros*)).ti,ab. 5242

6 war syndrome*.ti,ab. 309

7 (shell shock* or shellshock* or shell-shock*).ti,ab. 156

8 exp Psychological Trauma/ 1982

9 exp Stress Disorders, Traumatic/ 46034

10 exp Stress Disorders, Traumatic, Acute/ 541

11 ((traumatic or acute) adj stress disorder*).ti,ab. 16710

12 (railway spine or (rape adj2 trauma*) or reexperienc* or re experienc* or torture syndrome or traumatic neuros* or traumatic stress).ti,ab. 23156

13 (trauma* and (avoidance or grief or horror or death* or nightmare* or night mare* or emotion*)).ti,ab. 47697

14 (posttraumatic* or post traumatic* or post-traumatic* or stress disorder* or acute stress or asd or desnos or combat syndrome or concentration camp syndrome or extreme stress or flashback* or flash back* or hypervigilan* or hypervigilen* or psych* stress or psych* trauma* or psycho?trauma* or psychotrauma* or posttrauma* or traumagenic* or traumatic stress*).ti,ab. 143852

15 (sexual adj2 trauma).ti,ab. 1489

16 exp Sexual Trauma/ 133

17 or/1-16 199781

18 exp Eye Movement Desensitization Reprocessing/ 382

19 (EMDR or eye movement desensiti?ation reprocessing).tw. 799

20 "eye movement desensiti?ation and processing".tw. 4

21 18 or 19 or 20 871

22 17 and 21 685

23 (2018* or 2019* or 2020* or 2021* or 2022* or 2023*).dt. 8267307

24 22 and 23 324

**Embase**

Embase <1974 to 2023 Week 36>

1 exp posttraumatic stress disorder/ 80336

2 PTSD.ti,ab. 42375

3 moral* injur*.ti,ab. 732

4 exp combat stress/ 86

5 ((combat or battle or conflict or war or wars) adj5 (stress or disorder* or neuros*)).ti,ab. 6214

6 war syndrome*.ti,ab. 367

7 (shell shock* or shellshock* or shell-shock*).ti,ab. 154

8 exp psychotrauma/ 11517

9 exp acute stress disorder/ 1782

10 ((traumatic or acute) adj stress disorder*).ti,ab. 21855

11 (railway spine or (rape adj2 trauma*) or reexperienc* or re experienc* or torture syndrome or traumatic neuros* or traumatic stress).ti,ab. 30286

12 (trauma* and (avoidance or grief or horror or death* or nightmare* or night mare* or emotion*)).ti,ab. 67617

13 (posttraumatic* or post traumatic* or post-traumatic* or stress disorder* or acute stress or asd or desnos or combat syndrome or concentration camp syndrome or extreme stress or flashback* or flash back* or hypervigilan* or hypervigilen* or psych* stress or psych* trauma* or psycho?trauma* or psychotrauma* or posttrauma* or traumagenic* or traumatic stress*).ti,ab. 184551

14 (sexual adj2 trauma).ti,ab. 1996

15 exp sexual trauma/ 387

16 exp acute stress/ 7018

17 exp behavioral stress/ 1066

18 exp emotional stress/ 27866

19 exp critical incident stress/ 116

20 exp mental stress/ 201010

21 or/1-20 448749

22 exp "eye movement desensitization and reprocessing"/ 697

23 (EMDR or eye movement desensiti?ation reprocessing).tw. 1055

24 "eye movement desensiti?ation and processing".tw. 3

25 22 or 23 or 24 1331

26 21 and 25 1025

27 (2018* or 2019* or 2020* or 2021* or 2022* or 2023*).dc. 10765299

28 26 and 27 501

**PsycINFO**

APA PsycInfo <1806 to September Week 1 2023>

1 exp Posttraumatic Stress Disorder/ 40469

2 PTSD.ti,ab. 39343

3 moral* injur*.ti,ab. 720

4 ((combat or battle or conflict or war or wars) adj5 (stress or disorder* or neuros*)).ti,ab. 6166

5 war syndrome*.ti,ab. 90

6 (shell shock* or shellshock* or shell-shock*).ti,ab. 236

7 exp Acute Stress Disorder/ 681

8 exp Combat Experience/ 3319

9 exp Emotional Trauma/ 16356

10 exp Posttraumatic Stress/ 2124

11 exp Traumatic Neurosis/ 310

12 exp Trauma/ 81027

13 exp Psychological Stress/ 9773

14 exp Chronic Stress/ 3348

15 ((traumatic or acute) adj stress disorder*).ti,ab. 14129

16 (railway spine or (rape adj2 trauma*) or reexperienc* or re experienc* or torture syndrome or traumatic neuros* or traumatic stress).ti,ab. 22644

17 (trauma* and (avoidance or grief or horror or death* or nightmare* or night mare* or emotion*)).ti,ab. 32437

18 (posttraumatic* or post traumatic* or post-traumatic* or stress disorder* or acute stress or asd or desnos or combat syndrome or concentration camp syndrome or extreme stress or flashback* or flash back* or hypervigilan* or hypervigilen* or psych* stress or psych* trauma* or psycho?trauma* or psychotrauma* or posttrauma* or traumagenic* or traumatic stress*).ti,ab. 100318

19 (sexual adj2 trauma).ti,ab. 2310

20 exp Sexual Abuse/ 23241

21 or/1-20 210008

22 exp Eye Movement Desensitization Therapy/ 1917

23 (EMDR or eye movement desensiti?ation reprocessing).tw. 2239

24 "eye movement desensiti?ation and processing".tw. 4

25 22 or 23 or 24 2354

26 21 and 25 1680

27 limit 26 to yr="2018 -Current" 538

**Cochrane**

Search Name:

Date Run: 15/09/2023 15:05:55

Comment:

ID Search Hits

#1 MeSH descriptor: [Stress Disorders, Post-Traumatic] explode all trees 3689

#2 (PTSD):ti,ab,kw (Word variations have been searched) 5727

#3 moral* injur*:ti,ab,kw 204

#4 MeSH descriptor: [Combat Disorders] explode all trees 143

#5 ((combat or battle or conflict or war or wars) near/5 (stress or disorder* or neuros*)):ti,ab,kw 544

#6 war syndrome*:ti,ab,kw 165

#7 (shell shock* or shellshock* or shell-shock*):ti,ab,kw 9

#8 MeSH descriptor: [Psychological Trauma] explode all trees 134

#9 MeSH descriptor: [Stress Disorders, Traumatic] explode all trees 3882

#10 MeSH descriptor: [Stress Disorders, Traumatic, Acute] explode all trees 58

#11 MeSH descriptor: [Stress, Psychological] explode all trees 7999

#12 ((traumatic or acute) NEXT stress disorder*):ti,ab,kw 3050

#13 ("railway spine" or (rape near/2 trauma*) or reexperienc* or re experien* or "torture

syndrome" or traumatic neuros* or "traumatic stress"):ti,ab,kw 7327

#14 (trauma* and (avoidance or grief or horror or death* or nightmare* or night mare* or

emotion*)):ti,ab,kw 4758

#15 (posttraumatic* or post traumatic* or stress disorder* or "acute stress" or asd or

desnos or ("combat syndrome" or "concentration camp syndrome" or

"extreme stress" or flashback* or flash back* or hypervigilan* or hypervigilen* or psych*

stress or psych* trauma* or psychotrauma* or psychotrauma*) or (posttrauma* or

traumagenic* or traumatic stress*)):ti,ab,kw 43872

#16 (sexual NEAR/2 trauma):ti,ab,kw 119

#17 MeSH descriptor: [Sexual Trauma] explode all trees 13

#18 {OR #1-#17} 50803

#19 MeSH descriptor: [Eye Movement Desensitization Reprocessing] explode all trees 100

#20 (EMDR or eye movement desensiti?ation reprocessing):ti,ab,kw 579

#21 ("eye movement desensitization and processing" or "eye movement desensitization and processing"):ti,ab,kw 1

#22 #19 or #20 or #21 579

#23 #18 and #22 with Cochrane Library publication date Between Jan 2018 and Sep 2023 291

**CINAHL**

Accessibility Information and TipsPrint Search History

Friday, September 15, 2023 4:38:53 PM

# Query Limiters/Expanders Last Run Via Results

S22 S20 AND S21 Expanders - Apply equivalent subjects

Search modes - Boolean/Phrase Interface - EBSCOhost Research Databases

Search Screen - Advanced Search

Database - CINAHL 169

S21 Limiters - Published Date: 20180101-20231231

Expanders - Apply equivalent subjects

Search modes - Boolean/Phrase Interface - EBSCOhost Research Databases

Search Screen - Advanced Search

Database - CINAHL 2,564,970

S20 S15 AND S19 Expanders - Apply equivalent subjects

Search modes - Boolean/Phrase Interface - EBSCOhost Research Databases

Search Screen - Advanced Search

Database - CINAHL 404

S19 S16 OR S17 OR S18 Expanders - Apply equivalent subjects

Search modes - Boolean/Phrase Interface - EBSCOhost Research Databases

Search Screen - Advanced Search

Database - CINAHL 594

S18 "eye movement desensiti?ation and processing" Expanders - Apply equivalent subjects

Search modes - Boolean/Phrase Interface - EBSCOhost Research Databases

Search Screen - Advanced Search

Database - CINAHL 1

S17 (EMDR or eye movement desensiti?ation reprocessing) Expanders - Apply equivalent subjects

Search modes - Boolean/Phrase Interface - EBSCOhost Research Databases

Search Screen - Advanced Search

Database - CINAHL 485

S16 (MH "Eye Movement Desensitization and Reprogramming") Expanders - Apply equivalent subjects

Search modes - Boolean/Phrase Interface - EBSCOhost Research Databases

Search Screen - Advanced Search

Database - CINAHL 385

S15 S1 OR S2 OR S3 OR S4 OR S5 OR S6 OR S7 OR S8 OR S9 OR S10 OR S11 OR S12 OR S13 OR S14 Expanders - Apply equivalent subjects

Search modes - Boolean/Phrase Interface - EBSCOhost Research Databases

Search Screen - Advanced Search

Database - CINAHL 181,127

S14 (MH "Sexual Trauma+") OR (MH "Military Sexual Trauma") Expanders - Apply equivalent subjects

Search modes - Boolean/Phrase Interface - EBSCOhost Research Databases

Search Screen - Advanced Search

Database - CINAHL 119

S13 TI (sexual N2 trauma) OR AB (sexual N2 trauma) Expanders - Apply equivalent subjects

Search modes - Boolean/Phrase Interface - EBSCOhost Research Databases

Search Screen - Advanced Search

Database - CINAHL 1,136

S12 TI ( (posttraumatic* or post traumatic* or post-traumatic* or stress disorder* or acute stress or asd or desnos or combat syndrome or concentration camp syndrome or extreme stress or flashback* or flash back* or hypervigilan* or hypervigilen* or psych* stress or psych* trauma* or psycho?trauma* or psychotrauma* or posttrauma* or traumagenic* or traumatic stress*) ) OR AB ( (posttraumatic* or post traumatic* or post-traumatic* or stress disorder* or acute stress or asd or desnos or combat syndrome or concentration camp syndrome or extreme stress or flashback* or flash back* or hypervigilan* or hypervigilen* or psych* stress or psych* trauma* or psycho?trauma* or psychotrauma* or posttrauma* or traumagenic* or traumatic stress*) ) Expanders - Apply equivalent subjects

Search modes - Boolean/Phrase Interface - EBSCOhost Research Databases

Search Screen - Advanced Search

Database - CINAHL 63,498

S11 TI ( (trauma* and (avoidance or grief or horror or death* or nightmare* or night mare* or emotion*)) ) OR AB ( (trauma* and (avoidance or grief or horror or death* or nightmare* or night mare* or emotion*)) ) Expanders - Apply equivalent subjects

Search modes - Boolean/Phrase Interface - EBSCOhost Research Databases

Search Screen - Advanced Search

Database - CINAHL 17,332

S10 TI ( (railway spine or (rape N2 trauma*) or reexperienc* or re experienc* or torture syndrome or traumatic neuros* or traumatic stress) ) OR AB ( (railway spine or (rape N2 trauma*) or reexperienc* or re experienc* or torture syndrome or traumatic neuros* or traumatic stress) ) Expanders - Apply equivalent subjects

Search modes - Boolean/Phrase Interface - EBSCOhost Research Databases

Search Screen - Advanced Search

Database - CINAHL 12,281

S9 TI ( ((traumatic or acute) N1 stress disorder*) ) OR AB ( ((traumatic or acute) N1 stress disorder*) ) Expanders - Apply equivalent subjects

Search modes - Boolean/Phrase Interface - EBSCOhost Research Databases

Search Screen - Advanced Search

Database - CINAHL 7,493

S8 (MH "Stress, Psychological+") Expanders - Apply equivalent subjects

Search modes - Boolean/Phrase Interface - EBSCOhost Research Databases

Search Screen - Advanced Search

Database - CINAHL 103,646

S7 (MH "Psychological Trauma+") Expanders - Apply equivalent subjects

Search modes - Boolean/Phrase Interface - EBSCOhost Research Databases

Search Screen - Advanced Search

Database - CINAHL 2,737

S6 TI ( (shell shock* or shellshock* or shell-shock*) ) OR AB ( (shell shock* or shellshock* or shell-shock*) ) Expanders - Apply equivalent subjects

Search modes - Boolean/Phrase Interface - EBSCOhost Research Databases

Search Screen - Advanced Search

Database - CINAHL 61

S5 TI war syndrome* OR AB war syndrome* Expanders - Apply equivalent subjects

Search modes - Boolean/Phrase Interface - EBSCOhost Research Databases

Search Screen - Advanced Search

Database - CINAHL 127

S4 TI ( ((combat or battle or conflict or war or wars) N5 (stress or disorder* or neuros*)) ) OR AB ( ((combat or battle or conflict or war or wars) N5 (stress or disorder* or neuros*)) ) Expanders - Apply equivalent subjects

Search modes - Boolean/Phrase Interface - EBSCOhost Research Databases

Search Screen - Advanced Search

Database - CINAHL 2,279

S3 TI moral* injur* OR AB moral* injur* Expanders - Apply equivalent subjects

Search modes - Boolean/Phrase Interface - EBSCOhost Research Databases

Search Screen - Advanced Search

Database - CINAHL 454

S2 TI PTSD OR AB PTSD Expanders - Apply equivalent subjects

Search modes - Boolean/Phrase Interface - EBSCOhost Research Databases

Search Screen - Advanced Search

Database - CINAHL 14,419

S1 (MH "Stress Disorders, Post-Traumatic+") Expanders - Apply equivalent subjects

Search modes - Boolean/Phrase Interface - EBSCOhost Research Databases

Search Screen - Advanced Search

Database - CINAHL 27,213

**PTSDpubs**

MAINSUBJECT.EXACT.EXPLODE("EMDR") OR title(EMDR OR eye movement desensiti?ation reprocessing OR "eye movement desensiti?ation and processing") OR abstract(EMDR OR eye movement desensiti?ation reprocessing OR "eye movement desensiti?ation and processing")Limits applied (2018-2023)

Hand searching of key journals and websites Journal of EMDR Practice and Research, Current Approaches in Psychiatry, American Journal of Applied Psychology, Clinical Neuropsychiatry, Scientific Reports, European Journ- al of Therapeutics, Journal of Korean Neuropsychiatric Association, Psychology and Behavioral Science, Open Journal of Social Sciences, IberoAmerican Journal of Psychotrauma and Dissociation. Relevant organisation websites were searched: National Institute of Health and Care Excellence (NICE); American Psychological Association; EMDR Europe; EMDR Association of Australia; EMDR Institute of Israel.

# **Methods of data synthesis for clinical effectiveness**

In order for meta-analyses to be conducted, there needed to be at least three studies providing data for a comparison. Studies could be from our review, or the review on which NICE guidance was based (1). In order to be included in a meta-analysis, a study had to include both mean and SD for the change in PTSD from pre- to post-treatment, or these data had to be calculable.

Comparative effectiveness was evaluated using pairwise meta-analysis (MA).**(2)** Five comparisons were conducted, comparing direct evidence of treatment effect from multiple studies. The analyses were conducted using a Bayesian Markov Chain Monte Carlo (MCMC) approach using a random effects model to account for heterogeneity in treatment effects across studies.**(2)**

The outcome considered was the change in PTSD symptoms before and after treatment.

For the purposes of this review, positive change in mean indicated improvement, and negative change in mean indicated worsening of symptoms.

There were no possible meta-analyses for prevention, or for early treatment. All meta-analyses were for adults with PTSD given delayed treatment (i.e. three months or more following trauma). Pairwise meta-analyses were conducted for the following comparisons:

EMDR vs. TF-CBT, PTSD self-report, follow-up post-treatment;

EMDR vs. TF-CBT, PTSD clinician-rated, follow-up post-treatment;

EMDR vs. waitlist/usual care, PTSD self-report, follow-up post-treatment;

EMDR vs. waitlist/usual care, PTSD self-report, follow-up three months following treatment;

The scoring of PTSD symptoms was conducted using different scoring methods, studies reporting any validated PTSD scale were included. To enable the use of studies using different scoring methods within a single pairwise MA, standardised mean differences (SMDs) were calculated for each study. The use of SMDs is based on the assumption that all scoring scales are quantifying the same treatment effect and can be transformed onto a common scale by dividing the mean difference between the intervention and comparator within each study by the standard deviation of the difference. Raw data extracted from study results in the form of means/standard deviation/confidence intervals were used to evaluate the SMD and subsequently the standard error (SE) for each study using Hedge’s correction.**(3)** Using intervention 1 as the reference, the SMD for the interventions in arm $t=2$, at follow-up $f=1,2$ is given by

$${SMD}_{t,f}=c\cdot\frac{\mu_{f, t}-\mu_{f, 1}}{S}$$

$$S=\sqrt{\frac{{(n}_{1}-1)S_{1}^{2}+{(n}_{t}-1)S_{t}^{2}}{n_{1}+n_{t}-2}}$$

$$c=1-\frac{3}{4\left( n_{1}+n_{t} \right)-9}$$

where $\mu_{f, t}$ is the change in score before and after treatment for arm t at follow-up f; $S$ is the within group standard deviation pooled across groups and$c$ is Hedges’ correction factor. The standard error of the SMD is given by

$$SE({SMD}_{t,f})=\sqrt{c^{2}\left( \frac{n_{1}+n_{t}}{n_{1}n_{t}}+\frac{{{SMD}_{t,f}}^{2}}{2(n_{1}+n_{t})} \right)}$$

Where the 95% confidence intervals were provided as opposed to the standard deviation, the standard deviation was evaluated using,

$$SD=\sqrt{n}\frac{CI_{upper}-CI_{lower}}{C},$$

where $n$ is the number of particpants in the study arm, $CI_{upper}$ and $CI_{lower}$ are the upper and lower 95% confidence intervals respectively and $C$ was chosen to be 3.92 for all studies with n>60.**(3)** For studies which only reported pre- and post-treatment scores, the change in PTSD symptoms score before and after treatment was calculated based on the reported pre- and post-treatment scores and standard error was calculated assuming the correlation is 0.5.

## ***Statistical model for the meta-analysis***

Let $y_{ik}$ denote the SMD of arm $k$ of trial $i$ where $k=1,\ldots,na$ and $i=1,\ldots,ns$, with variance $V_{ik}$. We assume that the treatment effects are normally distributed such that,

$$y_{ik}\mathcal{\sim N}\left( \theta_{ik}, V_{ik} \right),$$

where $\boldsymbol{\theta}$ are the parameters of interest. The individual $\theta_{ik}$ are modelled using the identity link function as they are continuous and over the entire real line,

$$\theta_{ik}=\delta_{i,1k}.$$

To allow for heterogeneity of treatment effects across studies, a random effects model was assumed. The random effects model is structured such that all individual study treatment effects, $\delta_{i,1k}$ arise from a common normal distribution centred about a mean treatment effect with some variance, $\tau^{2}$,

$$\delta_{i,1k}\mathcal{\sim N(}d_{t_{i1}t_{ik}}, \tau^{2})$$

where $d_{t_{i1}t_{ik}}$ represents the mean effect of treatment $k$ of study $i$ ($t_{ik}$) compared to the treatment in arm 1 of study $i$($t_{i1}$).

As only pairwise meta-analyses were conducted, $k=1,2$, the subscripts within the model can be further simplified to the following,

$$y_{i2}\mathcal{\sim N}\left( \theta_{i2}, V_{i2} \right),$$

$$\theta_{i2}=\delta_{i,12},$$

$$\delta_{i,12}\mathcal{\sim N}\left( d_{t_{i1}t_{i2}}, \tau^{2} \right),$$

where $y_{i2}$ is the SMD between EMDR (subscript 2) and the comparator treatment (subscript 1) and $\delta_{i,12}$ is the study-specific treatment SMD between EMDR and the comparator.

Parameters were estimated using a Bayesian framework, as such non-informative priors were chosen for the between-study standard deviation of treatment effects, $\tau$, and the mean treatment effect, $d_{t_{i1}, t_{i2}}$:

- $\tau\sim\mathcal{U}\left( 0,5 \right)\cdot\frac{\sqrt{3}}{\pi}$
- $d_{t_{i1}, t_{i2}}\mathcal{\sim N(}{0,100}^{2})$

In cases where there was not sufficient evidence to inform inference on $\tau$, a more informative prior was chosen for the between study standard deviation of treatment effects,

$$\tau\sim\mathrm{lognormal}\left( 0.13, 0.249 \right).$$

This prior was derived using information from relevant comparisons with higher numbers of studies.

All analyses were conducted using the freely available software WinBUGS **(4)**via the R package, R2WinBUGS **(5).** Convergence to the target posterior was assessed using the Gelman-Rubin statistic.**(6)** All simulations appeared to converge within 20,000 iterations and so a burn-in period of 20,000 samples was chosen for all analyses. A further 10,000 iterations of the Markov chain were retained to estimate parameters of interest after thinning the chain samples by a factor of 10.

Results are presented alongside the posterior median treatment effects and 95% credible intervals (CrI). Effect sizes were graded using Cohen’s categories; not substantial (SMD<0.2), small (0.2$\leq$SMD<0.5), medium (0.5$\leq$SMD<0.8), large (0.8$\leq$SMD).**(7)** Study heterogeneity was graded and interpreted according to the categories introduced in Ren *et al.* 2018.(8)

**Table S1 Participant characteristics for included studies**

| **Author, year** | **Intervention** | **Single / multiple / mixed traumatic events** | **Type of trauma** | **Time since traumatic event in months (mean)** | **Time since PTSD onset in months (mean)** | **PTSD scale and score (Diagnostic status)** | **Age in years (mean)** | **Sex: female (%)** | **Ethnicity (%)** |
| --- | --- | --- | --- | --- | --- | --- | --- | --- | --- |
| **Adult, treatment, TF-CBT, delayed, PTSD clinician-report** | | | | | | | | | |
| Boterhoven 2020 / Assmann 2021 | EMDR | Mixed | Sexual abuse/ assault; physical abuse; Mixed abuse; Domestic violence; Serious injury/ death; other | NR | 218.07 | CAPS: 37.03 | 38.96 | 80.2 | European: 60.5  Australian: 28.4  African: 8.6  Asian: 2.5  Other: 2.5 |
|  | ImRs | Mixed |  | NR | 212.18 | CAPS: 38.61 | 38.08 | 73.0 | European: 52.7  Australian: 18.9  African: 14.9  Asian: 5.4;  Other: 4.1 |
| Santarnecchi 2019 | EMDR | Single | Natural disaster | 120 | 120 | CAPS: 57.6 | 28.6 | 58.8 | NR |
|  | TF-CBT | Single |  | 120 | 120 | CAPS: 45.7 | 26.3 | 64.3 | NR |
| Stanbury 2020 | EMDR | NR | Sexual abuse; domestic violence, motor vehicle accident; natural disaster; physical assault | Unclear (60% childhood trauma) | NR | PCL-C: 59 | 39.7 | NR | NR |
|  | PE | NR |  | Unclear (60% childhood trauma) | NR | PCL-C: 53.4 | 44.6 | NR | NR |
| **Adult, treatment, TF-CBT, delayed, PTSD self-report** | | | | | | | | | |
| Boterhoven 2020 / Assmann 2021 | EMDR | Mixed | Sexual abuse/ assault; physical abuse; Mixed abuse; Domestic violence; Serious injury/ death; other | NR | 218.07 | IES-R: 46.35 | 38.96 | 80.2 | European: 60.5  Australian: 28.4  African: 8.6  Asian: 2.5  Other: 2.5 |
|  | ImRs | Mixed |  | NR | 212.18 | IES-R: 46.66 | 38.08 | 73.0 | European: 52.7  Australian: 18.9  African: 14.9  Asian: 5.4;  Other: 4.1 |
| Greenwald, 2021 | EMDR | Multiple | Victims of crime | NR | NR | TSI-2†: Overall participants with data: 13.8. Data from author | Overall: 40.62 | Overall: 79.6 | Overall: White 84; multiracial 11; Hispanic 3; other 2 |
|  | PC | Multiple |  | NR | NR |  |  |  |  |
| Moghadam 2020 | EMDR | Multiple | Combat | Unclear | Unclear | Mississippi PTSD measure: 22.10 | NR | NR | NR |
|  | CBT |  |  |  |  | Mississippi PTSD measure: 26.93 | NR | NR | NR |
|  | No treatment |  |  |  |  | Mississippi PTSD measure: 33.97 | NR | NR | NR |
| Nijdam 2018 | EMDR | Single | Assault; Accident; Sexual assault; Disaster; War-related; Other | Unclear | Unclear | SI-PTSD: 38.3 | 41.6 | 55.3 | Dutch: 66 |
|  | BEP |  |  |  |  | SI-PTSD: 39.2 | 39.0 | 56.1 | Dutch: 63.4 |
| Stanbury 2020 | EMDR | NR | Sexual abuse; domestic violence, motor vehicle accident; natural disaster; physical assault | Unclear time since primary event. (60% childhood trauma) | NR | PCL-C: 59 | 39.7 | NR | NR |
|  | PE |  |  | Unclear time since primary event. (60% childhood trauma) | NR | PCL-C: 53.4 | 44.6 | NR | NR |
| Santarnecchi 2019 | EMDR | Single | Natural disaster | 120 | 120 | CAPS: 57.6 | 28.6 | 58.8 | NR |
|  | TF-CBT |  |  | 120 | 120 | CAPS: 45.7 | 26.3 | 64.3 | NR |
| **Adult, treatment, TF-CBT, early, PTSD self-report** | | | | | | | | | |
| Ironson 2021 | EMDR | Single | Overall: Violence 62.1%; Death 60.9%; Violent Death 27.6% | Overall 11.6 weeks (7.5) | NR | DTS: 57.55 | Overall: 46.21 | Overall: 60 | Overall: African American 77.1; Hispanic or Latino 11.4; other 11.5 |
|  | SMT |  |  | Overall 11.6 weeks (7.5) | NR | DTS: 55.48 |  |  |  |
|  | SC |  |  | Overall 11.6 weeks (7.5) | NR | DTS: 53.4 |  |  |  |
| **Adult, treatment, wait list/usual care, delayed, PTSD clinician-report** | | | | | | | | | |
| Zhao, 2023 | EMDR | Multiple | Overall: Domestic violence 49.1% | NR | NR | PCL-C: 65.0 | 25.5 | 82.1 | NR |
|  | WL |  |  | NR | NR | PCL-C: 65.5 | 24.6 | 86.2 | NR |
| **Adult, treatment, wait list/usual care, delayed, PTSD self-report** | | | | | | | | | |
| Moghadam 2020 | EMDR | Multiple | Combat | Unclear | Unclear | Mississippi PTSD measure: 22.10 | NR | NR | NR |
|  | CBT |  |  | Unclear | Unclear | Mississippi PTSD measure: 26.93 | NR | NR | NR |
|  | No treatment |  |  | Unclear | Unclear | Mississippi PTSD measure: 33.97 | NR | NR | NR |
| Zhao, 2023 | EMDR | Multiple | Overall: Domestic violence 49.1% | NR | NR | PCL-C: 65.0 | 25.5 | 82.1 | NR |
|  | Wait list |  |  | NR | NR | PCL-C: 65.5 | 24.6 | 86.2 | NR |
| Encinas, 2019 | EMDR-PRECI | NA | Parents of children ASD | NA | NA | PCL-5*: 37.35 | Overall 39.8 | Overall: 96 | NR |
|  | CAU |  |  | NA | NA | PCl-5*: 30.72 |  |  | NR |
| Farrell, 2023 | EMDR VGTEP | Unclear | Front Line Health, First responders and Emergency and Social Care Workers. | >6 months, n=2;  6-12 months, n=41;  >12 months, n=3 | NR | ITQ: Overall: 36.8 | 46.4 | 80 | NR |
|  | WL | Unclear |  | >6 months, n=10;  6-12 months, n=27;  >12 months, n=2 | NR |  | 45.5 | 77 |  |
| Jarero, 2018 | EMDR IGPT-OTS | Ongoing | In the active or follow-up phase of cancer treatment | Overall: Range: 3-54 months | NR | PCL-5*: 44.89 | Overall 47.02 | 100 | NR |
|  | No treatment |  |  |  |  | PCl-5*: 43.85 |  | 100 |  |
| Jarero, 2019 | EMDR-PRECI | Unclear | First responder exposed to wide variety of trauma | NR | NR | PCL-5*: 37.35 | 36.53 | 30.0 | NR |
|  | No treatment |  |  | NR | NR | PCl-5*: 30.72 | 30.93 | 33.3 |  |
| Rousseau 2019 | EMDR | Single | Accident 4; holdup 1; physical assault 7 | NR | 12 months (365 days) | PCL-C: 59.50 | 45.25 | 50.0 | NR |
|  | WL/ supportive therapy |  | Accident 3; holdup 2; physical assault 7 | NR | 14 months (426 days) | PCL-C: 60.75 | 40.08 | 58.3 | NR |
| **Adult, treatment, wait list/usual care, early, PTSD self-report** | | | | | | | | | |
| Perez 2020 | EMDR-IGTP | Multiple (ongoing) | Healthcare Professionals Working in Hospitals during Covid-19 Pandemic | Ongoing | Unclear: "seven weeks after the first Covid-19 patients died" | PCL-5: 35.85 | NR | NR | NR |
|  | WL |  |  |  |  | PCL-5: 35.48 | NR | NR | NR |
| Shapiro 2018 | EMDR R-TEP | Multiple (ongoing) | War-related trauma | <3 months | NR | PCL-5: 46.4 | 41.7 | 84.6 | NR |
|  | WL |  |  | <3 months | NR | PCL-5: 38.8 | 36.2 | 91.7 | NR |
| Ironson 2021 | EMDR | Single | Overall: Violence 62.1%; Death 60.9%; Violent Death 27.6% | Overall 11.6 weeks (7.5) | NR | DTS: 57.55 | Overall: 46.21 | Overall: 60 | Overall 77.1% African American; 11.4% Hispanic or Latino; 11.5% other |
|  | SMT | Single |  |  | NR | DTS: 55.48 |  |  |  |
|  | SC (PFA) | Single |  |  | NR | DTS: 53.4 |  |  |  |
| **Adult, prevention, wait list/usual care, delayed, PTSD self-report** | | | | | | | | | |
| Bates, 2023 | EMDR | Single | COVID-19 hospitalisation | NR | NR | PCL-C: Overall: 29.2 | 58.3 | 38.5 | White British: 12 (92.3);  White other: 1 (7.7);  Unknown: 0 (0) |
|  | CAU | Single |  | NR | NR | PCL-C: Overall: 29.2 | 57.7 | 38.5 | White British: 11 (92.3);  White other: 1 (7.7);  Unknown: 1 (7.7) |
| Farrell, 2023 | EMDR | Unclear | Front Line Health, First responders and Emergency and Social Care Workers; Impact of Events Scale Revised Score of 24 and above. | >6 months, n=2;  6-12 months, n=41;  >12 months, n=3 | NR | ITQ: Overall: 36.8 | 46.4 | 80 | NR |
|  | WL | Unclear |  | >6 months, n=10;  6-12 months, n=27;  >12 months, n=2 |  |  | 45.5 | 77 | NR |

BEP: Brief Eclectic Psychotherapy; CAPS: Clinician Administered PTSD Scale; CAU: Care as usual; CBT: Cognitive Behavioural Therapy; DTS: Davidson Trauma Scale; EMDR: Eye Movement and Desensitisation Reprocessing; IGTP-OTS: Integrative Group Treatment Protocol-Ongoing Traumatic Stress; ImRs: Imagery Rescripting; ITQ: International Trauma Questionnaire; NA: Not applicable; NR: Not reported; PCL-C: PTSD Checklist for DSM-5 (PCL-5) Civilian version; PC: Progressive Counting; PE: Prolonged Exposure; PFA: Psychological First Aid; PRECI: Protocol for Recent Critical Incidents and Ongoing Traumatic Stress PTSD: Post Traumatic Stress Disorder; RCT: Randomised Controlled Trial; R-TEP: Recent Traumatic Episode Protocol; SC: Standard Care; TSI-2: SMT: Stress Management with a Trauma focus; TF-CBT: Trauma-focused CBT; TSI-2: Trauma Symptom Inventory 2; VGTEP: Video-conference Group Traumatic Episode Protocol; WL: Waitlist. Early (within three months of event); Delayed (more than three months after event)

**PTSD results tables**

Note: Some studies are presented in tables more than once as they provided data for more than one comparison.

**Table S2 Adult, treatment, TF-CBT, delayed, PTSD self-report**

| **Author, year** | **TF-CBT type** | **PTSD self-report scale used** | **Follow-up** | **EMDR, n** | **EMDR baseline mean** | **EMDR baseline SD** | **EMDR mean change from baseline** | **EMDR SD change from baseline** | **TF-CBT, n** | **TF-CBT baseline mean** | **TF-CBT baseline SD** | **TF-CBT mean change from baseline** | **TF-CBT SD change from baseline** | **EMDR versus TF-CBT treatment group x time interaction** |
| --- | --- | --- | --- | --- | --- | --- | --- | --- | --- | --- | --- | --- | --- | --- |
| BOTERHOVEN 2020 and ASSMANN 2021 | imagery rescripting | IES-R (All traumas) | post-treatment | 81 | 46.35 | 95% CI: (42.45–50.59) | 24.36 | NR | 74 | 46.66 | 95% CI: (42.60–51.09) | 22.55 | NR | P= 0.52 |
| BOTERHOVEN 2020 and ASSMANN 2021 | imagery rescripting | IES-R (All traumas) | 1 year | 66 | 46.35 | 95% CI: (42.45–50.59) | 23.42 | NR | 68 | 46.66 | 95% CI: (42.60–51.09) | 27.07 | NR | NR |
| Greenwald, 2021† | Intensive Progressive Counting | TSI-2, number of clinically significant scales | post-treatment | 10 | 13.5 | NR | 10.5 | NR | 12 | 9.1 | NR | 7.2 | NR | NR |
| Greenwald, 2021† | Intensive Progressive Counting | TSI-2, number of clinically significant scales | 12 weeks | 13 | 15 | NR | 10.7 | NR | 20 | 13.75 | NR | 8.9 | NR | NR |
| Greenwald, 2021† | Intensive Progressive Counting | Problem rating scale | post-treatment | 10 | 6.3 | NR | 4.3 | NR | 16 | 6.7 | NR | 4.7 | NR | NR |
| Moghadam 2020 | CBT | Mississippi PTSD measure | post-treatment | 15 | 22.1 | 9.08 | 1.66 | 8.68 | 15 | 26.93 | 5.86 | 0.47 | 8.44 | P=<0.01 sig favours EMDR |
| Stanbury 2020 | prolonged exposure | PCL-C | 6 weeks | 10 | 59 | 16.63 | 24.6 | 16.9 | 10 | 53.4 | 12.55 | 18.5 | 13.52 | non-significant |
| Stanbury 2020 | prolonged exposure | PCL-C | 6 months | 7 | 59 | 16.63 | 25.3 | 17.41 | 8 | 53.4 | 12.55 | 25.3 | 11.99 | NR |
| Nijdam 2018 | brief eclectic psychotherapy | IES-R | 17 weeks | 47 | 69.9 | 21.3 | NR | NR | 41 | 81.3 | 14.1 | NR | NR | p = 0.48 |
| Santarnecchi 2019 | TF-CBT | DTS total score | post-treatment (EMDR average 4 weeks, TF-CBT average 10 weeks) | 17 | 45 | NR | 14 | NR | 14 | 38 | NR | 12 | NR | p=0.96 |

†Information provided in communication from authors, results from treatment completers providing data at two time points

BEP: Brief Eclectic Psychotherapy; CAPS: Clinician Administered PTSD Scale; CAU: Care as usual; CBT: Cognitive Behavioural Therapy; DTS: Davidson Trauma Scale; EMDR: Eye Movement and Desensitisation Reprocessing; IES-R: Impact of Events Scale-revised; IGTP-OTS: Integrative Group Treatment Protocol-Ongoing Traumatic Stress; ImRs: Imagery Rescripting; ITQ: International Trauma Questionnaire; NA: Not applicable; NR: Not reported; PCL-5: PTSD Checklist for DSM-5; PCL-C: PTSD Checklist for DSM-5 Civilian version; PE: Prolonged Exposure; PFA: Psychological First Aid; PRECI: Protocol for Recent Critical Incidents and Ongoing Traumatic Stress PTSD: Post Traumatic Stress Disorder; RCT: Randomised Controlled Trial; R-TEP: Recent Traumatic Episode Protocol; SC: Standard Care; TSI-2: SMT: Stress Management with a Trauma focus; TF-CBT: Trauma-focused CBT; TSI-2: Trauma Symptom Inventory 2; VGTEP: Video-conference Group Traumatic Episode Protocol; WL: Waitlist.

**Table S3 Adult, treatment, TF-CBT, delayed PTSD clinician-report**

| **Author, year** | **TF-CBT type** | **PTSD clinician-report scale used** | **Follow-up** | **EMDR, n** | **EMDR baseline mean** | **EMDR baseline SD** | **EMDR mean change from baseline** | **EMDR SD change from baseline** | **TF-CBT, n** | **TF-CBT baseline mean** | **TF-CBT baseline SD** | **TF-CBT mean change from baseline** | **TF-CBT SD change from baseline** | **EMDR versus TF-CBT treatment group x time interaction** |
| --- | --- | --- | --- | --- | --- | --- | --- | --- | --- | --- | --- | --- | --- | --- |
| BOTERHOVEN 2020 and ASSMANN 2021 | imagery rescripting | CAPS-5 | post-treatment | 81 | 37.03 | 95% CI: (34.23–40.06) | 15.62 | NA | 74 | 38.61 | 95% CI: (35.65–41.82) | 14.34 | NA | P=0.96 |
| BOTERHOVEN 2020 and ASSMANN 2021 | imagery rescripting | CAPS-5 | 1 year | 81 | 37.03 | 95% CI: (34.23–40.06) | 19.07 | NA | 74 | 38.61 | NA | 22.43 | NA | NR |
| Stanbury 2020 | PE | CAPS | 6 weeks | 10 | 86.71 | 22.85 | 64.14 | 24.41 | 10 | 77.88 | 13.07 | 60.75 | 16.26 | non-significant |
| Stanbury 2020 | PE | CAPS | 6 months | 7 | 86.71 | 22.85 | 48.14 | 25.61 | 8 | 77.88 | 13.07 | 53.5 | 13.31 | NR |
| Santarnecchi 2019 | TF-CBT | CAPS | post-treatment (EMDR average 4 weeks, TF-CBT average 10 weeks) | 17 | 57.6 | NR | 27.6 | NR | 14 | 45.7 | NR | 36.7 | NR | p=0.9 |

CAPS: Clinician Administered PTSD Scale; CAPS-5: Clinician Administered PTSD Scale for DSM5; CBT: Cognitive Behavioural Therapy; EMDR: Eye Movement and Desensitisation Reprocessing; ImRs: Imagery Rescripting; NA: Not applicable; NR: Not reported; PTSD: Post Traumatic Stress Disorder; RCT: Randomised Controlled Trial; R-TEP: Recent Traumatic Episode Protocol; SC: Standard Care; TSI-2: SMT: Stress Management with a Trauma focus; TF-CBT: Trauma-focused CBT;

**Table S4 Adult, treatment, TF-CBT, early, PTSD self-report**

| **Author, year** | **TF-CBT type** | **PTSD self-report scale used** | **Follow-up** | **EMDR, n** | **EMDR baseline mean** | **EMDR baseline SD** | **EMDR mean change from baseline** | **EMDR SD change from baseline** | **TF-CBT, n** | **TF-CBT baseline mean** | **TF-CBT baseline SD** | **TF-CBT mean change from baseline** | **TF-CBT SD change from baseline** | **EMDR versus TF-CBT treatment group x time interaction** |
| --- | --- | --- | --- | --- | --- | --- | --- | --- | --- | --- | --- | --- | --- | --- |
| Ironson, 2021 | Group-administered Stress Management with a  Trauma Focus | DTS | 1 month | 25 | 57.55 | NR | 26.37 | NR | 29 | 55.48 | NR | 22.81 | NR | p =0.131 [across three treatment groups] |
| Ironson, 2021 | Group-administered Stress Management with a  Trauma Focus | DTS | 6 months | 22 | 57.55 | NR | 25.11 | NR | 29 | 55.48 | NR | 24.69 | NR |  |

DTS: Davidson Trauma Scale; EMDR: Eye Movement and Desensitisation Reprocessing

**Table S5 Adult, treatment, wait-list/usual care, delayed, PTSD self-report**

(Note that one study from the NICE evidence (Jensen 1994) was excluded from the meta-analysis, because of an error in the NICE figure which used post-test M-PTSD, not the standardised mean difference.(1, 9)

| **Author, year** | **comparator** | **PTSD self-report scale used** | **Follow-up** | **EMDR, n** | **EMDR baseline mean** | **EMDR baseline SD** | **EMDR mean change from baseline** | **EMDR SD change from baseline** | **comparator, n** | **comparator baseline mean** | **comparator baseline SD** | **comparator mean change from baseline** | **comparator SD change from baseline** | **EMDR versus comparator treatment group x time interaction** |
| --- | --- | --- | --- | --- | --- | --- | --- | --- | --- | --- | --- | --- | --- | --- |
| Encinas, 2019 | Care as usual | PCL-5 | 3 days | 14 | 37.35 | 11.92 | 24.61 | 11.63 | 12 | 30.72 | 18.52 | Minus 3.18 | 19.35 | between groups at follow-up p <.01 |
| Encinas, 2019 | Care as usual | PCL-5 | 3 months | 14 | 37.35 | 11.92 | 25.83 | 12.12 | 12 | 30.72 | 18.52 | Minus4.09 | 18.96 | between groups at follow-up p <.01 |
| Farrell, 2023 | Delayed intervention | ITQ | Post-treatment | 46 | NR | NR | NR | NR | 39 | NR | NR | NR | NR | p < 0.001 |
| Jarero, 2018 | No treatment control | PCL-5 | 2 days | 35 | 44.89 | 10.26 | 24.38 | 10.51 | 26 | 43.85 | 11.19 | 6.66 | 13.27 | p < .001 |
| Jarero, 2018 | No treatment control | PCL-5 | 3 months | 35 | 44.89 | 10.26 | 27.0 | 12.07 | 26 | 43.85 | 11.19 | 8.75 | 11.55 | p < .001 |
| Jarero, 2019 | No treatment control | PCL-5 | 1 day | 30 | 39.2 | 8.02 | 35.9 | 7.39 | 30 | 38.27 | 6.22 | 5.57 | 7.35 | between groups at follow-up p < .01, |
| Moghadam 2020 | No treatment control | Unclear, but based on Mississippi PTSD measure | Post-treatment | 15 | 22.1 | 9.08 | 1.66 | 8.68 | 15 | 33.67 | 11.81 | 1.23 | 13.51 | P<0.01 sig favours EMDR |
| Rousseau 2019 | wait list, supportive therapy | PCL | Post-treatment | 12 | 59.5 | 12.93 | 31.09 | 12.12 | 12 | 60.75 | 13.05 | 7.17 | 18.07 | p < .001 favours EMDR |
| Zhao 2023 | wait-list. | PCL-C | 3 months | 28 | 65 | 11 | 18.9 | 15.83 | 29 | 65.5 | 9.3 | 6.4 | 11.27 | p<0.001, sig favours EMDR |

CAPS: Clinician Administered PTSD Scale; CAU: Care as usual; DTS: Davidson Trauma Scale; EMDR: Eye Movement and Desensitisation Reprocessing; ITQ: International Trauma Questionnaire; NA: Not applicable; NR: Not reported; PCL-C: PTSD Checklist for DSM-5 (PCL-5) Civilian version; PTSD: Post Traumatic Stress Disorder; RCT: Randomised Controlled Trial; TSI-2: Trauma Symptom Inventory 2; WL: Waitlist.

**Table S6 Adult, treatment, wait-list/usual care, delayed, PTSD clinician-report**

| **Author, year** | **comparator** | **PTSD clinician report scale used** | **Follow-up** | **EMDR, n** | **EMDR baseline mean** | **EMDR baseline SD** | **EMDR mean change from baseline** | **EMDR SD change from baseline** | **comparator, n** | **comparator baseline mean** | **comparator baseline SD** | **comparator mean change from baseline** | **comparator SD change from baseline** | **EMDR versus comparator treatment group x time interaction** |
| --- | --- | --- | --- | --- | --- | --- | --- | --- | --- | --- | --- | --- | --- | --- |
| Zhao 2023 | waitlist | CAPS | 3 months | 28 | 75 | 13.7 | 34.2 | 20.59 | 29 | 74 | 13.8 | 11.5 | 15.06 | p<0.001, favours EMDR |

CAPS: Clinician Administered WL: Waitlist.

**Table S7 Adult, treatment, wait-list/usual care, early, PTSD self-report**

| **Author, year** | **comparator** | **PTSD self-report scale used** | **Follow-up** | **EMDR, n** | **EMDR baseline mean** | **EMDR baseline SD** | **EMDR mean change from baseline** | **EMDR SD change from baseline** | **comparator, n** | **comparator baseline mean** | **comparator baseline SD** | **comparator mean change from baseline** | **comparator SD change from baseline** | **EMDR versus comparator treatment group x time interaction** |
| --- | --- | --- | --- | --- | --- | --- | --- | --- | --- | --- | --- | --- | --- | --- |
| Perez 2020 | wait list | PCL-C | 15 days post EMDR (or waitlist) | 35 | 35.85 | 6.42 | 14.63 | 6.48 | 39 | 35.48 | 3.64 | minus2.16 | 5.1 | p <0.001 favours EMDR |
| Shapiro 2018 | wait list | PCL-5 | post treatment | 10 | 50.5 | 12.4 | 21.9 | 8.5 | 6 | 32 | 6.3 | minus 6.2 | 2.1 | NR |
| Shapiro 2018 | wait list | PCL-5 | 6 months | 10 | 50.5 | 12.4 | 27.4 | 2.5 | 6 | 32 | 6.3 | 1.2 | 12.5 | NR |
| Ironson, 2021 | Standard of care (SC) | Davidson | 1 month | 25 | 57.55 | NR | 26.37 | NR | 30 | 53.40 | NR | 19.24 | NR | NR |
| Ironson, 2021 | Standard of care (SC) | Davidson | 6 months | 22 | 57.55 | NR | 25.11 | NR | 29 | 53.40 | NR | 24.26 | NR | p =0.131 [across three treatment groups] |

PCL-5: PTSD Checklist for DSM-5; PCL-C: PTSD Checklist Civilian version

**Table S8 Adult, prevention, wait-list/usual care, delayed, PTSD self-report**

| **Author, year** | **comparator** | **PTSD self-report scale used** | **Follow-up** | **EMDR, n** | **EMDR baseline mean** | **EMDR baseline SD** | **EMDR mean change from baseline** | **EMDR SD change from baseline** | **comparator, n** | **comparator baseline mean** | **comparator baseline SD** | **comparator mean change from baseline** | **comparator SD change from baseline** | **EMDR versus comparator treatment group x time interaction** |
| --- | --- | --- | --- | --- | --- | --- | --- | --- | --- | --- | --- | --- | --- | --- |
| Bates 2023 | Usual care | PCL-C | 6 months | 11 | 29.2 | Not reported | 8 | 10.49 | 12 | Not reported | Not reported | minus 0.75 | 15.17 | NR |

PCL-C: PTSD Checklist Civilian version

**Discontinuation rates**

Acceptability of treatments is not reported in any trials, but discontinuation of treatment (dropouts or withdrawals during the treatment period) might act as a proxy for this outcome (Supplementary Table S9).

Discontinuation rates in the trials with more intensive or shorter duration of EMDR were generally very low: either, very frequently, with no drop-outs, for both EMDR and comparator therapies or arms (10-15); or only a single drop-out in the EMDR arm (16), all indicating potentially high acceptability of the intervention. One trial in frontline workers during the COVID-19 Pandemic had drop-outs of 8% and 13.3% in the EMDR and WL groups, respectively(17). One study with a 4-week treatment period reported no discontinuation in the EMDR arm (n=34)(18). As treatment duration increased to 6 weeks or more, discontinuation rates increased (for all therapies). In four trials with EMDR treatment periods of between 6 and 12 weeks (19) (20-22) (n=320), the discontinuation rates for any EMDR arm were reported as 0% (vs 0% in PE control group(21)), 6.4% (vs 14.6% in the much longer duration BEP group(20)), 7.4% (vs 8.4% in the ImRs group(19)) at 6-8 weeks, and 17.9% at 12 weeks (vs 0% in the waitlist group(22)). The evidence indicates that EMDR as a therapy has a low discontinuation rate which is generally comparable to or slightly better than some comparator therapies, with the caveat that these studies have relatively small sample sizes. Trials may have lower discontinuation than in practice, however, given the brief duration of therapy it is expected that the risk of discontinuation will be low.

**Table S9 Discontinuations**

| **Author, year** | **Interventions** | **Treatment duration (as reported)** | **Treatment Discontinuations, n/N (%)** | **Study discontinuations** |
| --- | --- | --- | --- | --- |
| **Adult, treatment, TF-CBT, delayed** | | | | |
| Boterhoven 2020, Assmann 2021 | EMDR | 6-8 weeks | 6 / 81 (7.4) | 6/81 (7.4) |
|  | ImRs | 6-8 weeks | 6 / 74 (8.1) | 6/74 (8.1) |
| Greenwald, 2021 | Intensive EMDR | 2.5 to 5 days (mean 30.70 [SD = 17.79] hours of treatment) | 4/28 (14.3) | 10/24† (of the treatment completers, provided data post treatment or follow-up) |
|  | PC |  | 2/32 (6.3) | 5/30 |
| Moghadam 2020 | EMDR | Unclear (4 sessions) | 0/15 (0) | 0/15 (0) |
|  | TF-CBT | Unclear (8 sessions) | 0/15 (0) | 0/15 (0) |
| Nijdam 2018 | EMDR | Mean 6.4 (SD = 3.8) weekly sessions of 90 min | 3/47 (6.4) | 3/47 (6.4) |
|  | BEP | Mean 14.7 (SD = 4.5) weekly sessions of 45 min | 6/41 (14.6) | 6/41 (14.6) |
| Santarnecchi 2019 | EMDR | Average of 4 weeks (±2) of weekly sessions | 0/17 (0) | 0/17 (0) |
|  | TF-CBT | Average of 10 weekly visits (±2) | 0/14 (0) | 0/14 (0) |
| Stanbury 2020 | EMDR | 6 weeks | 0/10 (0) | 3/10 |
|  | Prolonged Exposure (PE) | 6 weeks | 0/10 (0) | 2/10 |
| **Adult, treatment, TF-CBT, early** | | | |  |
| Ironson, 2021 | EMDR | 4 weeks | 0/34 (0) | 0/34 (0) |
|  | Group administered SMT | 4 weeks | NR/37 (NR) | NR/37 (NR) |
| **Adult, treatment, wait list/usual care, delayed** | | | |  |
| Encinas, 2019 | EMDR-PRECI | 3 days (Six 1-hour individual treatment sessions, twice daily during three consecutive days) | 0/14 (0) | 0/14 (0) |
|  | Care as usual | NR | 0/12 (0) | 0/12 (0) |
| Farrell, 2023 | EMDR- VGTEP | 1 week | 4/50 (8) | 4/50 (8) |
|  | WL | 4 weeks | NA | 6/45 (13.3) |
| Jarero, 2018 | EMDR-IGPT-OTS | 2 days (six treatment sessions during 2 consecutive days, three times daily) | 0/35 (0) | 0/35 (0) |
|  | No treatment | NA | NA | 9/35 |
| Jarero, 2019 | EMDR-PRECI | 1 day (Two 1-hour individual treatment sessions on the same day) | 0/30 (0) | 0/30 (0) |
|  | No treatment | NA | NA | 0/30 (0) |
| Moghadam 2020 | EMDR | Unclear (4 sessions) | 0/15 (0) | 0/15 (0) |
|  | No treatment | NA | NA | 0/15 (0) |
| Rousseau 2019 | EMDR | One-hour sessions every 7–15 days; mean duration of therapy, 2.83 (SD =0.38) hours | NR/18 | 6/18 |
|  | WL/supportive therapy | One-hour sessions every 7–15 days; mean duration of therapy, 2.75 (SD=0.45) hours | NR/18 | 6/18 |
| Zhao 2023 | EMDR | 12 weeks (weekly 90-minute sessions) | 5/28 (17.9) | 5/28 (17.9) |
|  | WL | NA | NA | 0/29 (0) |
| **Adult, treatment, wait list/usual care, early** | | | |  |
| Perez 2020 | EMDR-IGTP | 1 week (four online group treatment sessions provided once a day during interspersed days (i.e., Monday, Wednesday, Friday, Sunday) | 0/40† | 5/40 |
|  | WL | NA | NA | 1/40 |
| Shapiro 2018 | EMDR | 3 days (three 90-minute sessions on consecutive days) | 1/13 (7.7) | 1/13 (7.7) |
|  | WL | NA | NA | 0/12 (0) |
| Ironson, 2021 | EMDR | 4 weeks | 0/34 (0) | 0/34 (0) |
|  | Group-administered Psychological First Aid (PFA) | 4 weeks | NR/37 (NR) | NR/37 (NR) |
| **Adult, prevention, wait list/usual care, delayed** | | | |  |
| Bates, 2023 | EMDR (online) | 1-8 weeks (Up to 8 weekly sessions; mean 3.25 sessions per participant) | 1/12 (8.3) | 2/13 |
|  | Care as usual | NR | 0/13 (0) | 1/13 |
| Farrell, 2023 | EMDR- VGTEP | 1 week | 4/50 (8) | 4/50 (8) |
|  | WL | NA | NA | 6/45 (13.3) |

BEP: Brief Eclectic Psychotherapy; CAPS: Clinician Administered PTSD Scale; CAU: Care as usual; CBT: Cognitive Behavioural Therapy; DTS: Davidson Trauma Scale; EMDR: Eye Movement and Desensitisation Reprocessing; IGTP-OTS: Integrative Group Treatment Protocol-Ongoing Traumatic Stress; ImRs: Imagery Rescripting; ITQ: International Trauma Questionnaire; NA: Not applicable; NR: Not reported; PCL-C: PTSD Checklist for DSM-5 (PCL-5) Civilian version; PC: Progressive Counting; PE: Prolonged Exposure; PFA: Psychological First Aid; PRECI: Protocol for Recent Critical Incidents and Ongoing Traumatic Stress PTSD: Post Traumatic Stress Disorder; RCT: Randomised Controlled Trial; R-TEP: Recent Traumatic Episode Protocol; SC: Standard Care; TSI-2: SMT: Stress Management with a Trauma focus; TF-CBT: Trauma-focused CBT; TSI-2: Trauma Symptom Inventory 2; VGTEP: Video-conference Group Traumatic Episode Protocol; WL: Waitlist.

Adverse events potentially associated with EMDR and the other therapies evaluated here were very rare in adults (Table S10).

**Table S10 Adverse events**

| **Author, year** | **Interventions (n)** | **Follow-ups** | **Adverse events** |
| --- | --- | --- | --- |
| **Adult, treatment, TF-CBT, delayed** | | | |
| Boterhoven 2020, Assmann 2021 | EMDR (81) vs ImRs (74) | Post-treatment, 8 weeks,  1 year | ‘Four serious adverse events were reported, with two of these deemed by participants as being partly study related. Both participants reported an increase in PTSD symptoms and suicidal ideation resulting in psychiatric admission, one after session six and the other after session twelve, which occurred after experiencing another trauma (road accident). Of the two who reported adverse events that were not study related, one had an in-patient admission after  a long-term relationship break-up after the wait-list assessment but before the start of treatment, and the other was admitted to hospital after losing their job, 4 months after completing treatment’. |
| Greenwald, 2021 | Intensive EMDR (24) vs PC (30) | 12 weeks | ‘Several participants reported brief periods of increased symptoms. This occurred in two distinct circumstances. The most common was when treatment had been only partially completed and there was a delay of several days to several weeks prior to having been able to schedule the rest of it; a few participants in that situation reported an increase of intrusive symptoms, reactivity, and/or general malaise. Following completion of treatment, a couple of participants also reported what we call ‘‘passing storms’’ of a brief period (perhaps a day or several) of feeling ‘‘messed-up,’’ hyper-sensitive, etc. Unfortunately, we did not formally track these reports.’ |
| **Adult, treatment, wait list/usual care, delayed** | | | |
| Encinas, 2019 | EMDR-PRECI (14) vs CAU (12) | 90 days (post treatment) | No adverse effects were reported during treatment or at 90-day follow-up. |
| Jarero, 2018 | EMDR-IGPT-IOS (35) vs no treatment (30) | 90 days (post treatment) | No adverse effects were reported during treatment or at 90-day follow-up. |
| Jarero, 2019 | EMDR-PRECI (30) vs no treatment (30) | 90 days (post treatment) | No adverse effects were reported during treatment or at 90-day follow-up. |
| Zhao 2023 | EMDR (28) vs WL (29) | 12 weeks (post treatment) | ‘Three patients (5.3 %) reported suicide attempts during the study, one in the EMDR group and two in the WL group (3.6 % vs.6.9 %, *P* = 1.000). No other severe adverse events (SAEs) were reported’. |
| **Adult, treatment, wait list/usual care, early** | | | |
| Perez 2020 | EMDR-IGTP (40) vs WL (40) | Post-treatment and 90 days  after WL treatment completion | ‘No adverse effects (e.g., symptoms of dissociation, fear, panic, freeze, shut down, collapse, fainting), or events (e.g., suicide ideation, suicide attempts, self-harm, homicidal ideation) were reported by the participants during treatment or at three months post-treatment follow-up’. |
| **Adult, prevention, wait list/usual care, delayed** | | | |
| Bates, 2023 | EMDR (online) (12) vs CAU (13) | 6 months | No adverse events were reported. |

BEP: Brief Eclectic Psychotherapy; CAPS: Clinician Administered PTSD Scale; CAU: Care as usual; CBT: Cognitive Behavioural Therapy; DTS: Davidson Trauma Scale; EMDR: Eye Movement and Desensitisation Reprocessing; IGTP-OTS: Integrative Group Treatment Protocol-Ongoing Traumatic Stress; ImRs: Imagery Rescripting; ITQ: International Trauma Questionnaire; NA: Not applicable; NR: Not reported; PCL-C: PTSD Checklist for DSM-5 (PCL-5) Civilian version; PC: Progressive Counting; PE: Prolonged Exposure; PFA: Psychological First Aid; PRECI: Protocol for Recent Critical Incidents and Ongoing Traumatic Stress PTSD: Post Traumatic Stress Disorder; RCT: Randomised Controlled Trial; R-TEP: Recent Traumatic Episode Protocol; SC: Standard Care; TSI-2: SMT: Stress Management with a Trauma focus; TF-CBT: Trauma-focused CBT; TSI-2: Trauma Symptom Inventory 2; VGTEP: Video-conference Group Traumatic Episode Protocol; WL: Waitlist.

Depression or anxiety (Table S11) were measured in 12 trials: EMDR had similar effectiveness to TF-CBT(19) (18, 21). Six studies of delayed treatment found EMDR significantly improved depression more than waitlist or usual care(12-14) (17, 22, 23) , and five studies found a significant benefit for EMDR over waitlist or usual care for anxiety (12-14) (17, 22). In early treatment or prevention, EMDR had a significant advantage over waitlist or usual care for depression(15, 16, 18, 24) and anxiety (15) (24)).

**Table S11 Depression and anxiety**

| **Author, year** | **Interventions (n)** | **Follow-ups** | **Depression**  Mean change from baseline / pre-therapy (estimated 95% CI) | **Anxiety** |
| --- | --- | --- | --- | --- |
| **Adult, treatment, TF-CBT, delayed** | | | | |
| Boterhoven 2020, Assmann 2021 | EMDR (81) vs ImRs (74) | Post-treatment, 8 weeks,  1 year | BDI II: From baseline to post-treatment, 8 weeks and 1 year  EMDR: 14.09 (NR), 14.06 (NR), 14.01 (NR)  ImRs: 10.55 (NR), 12.48 (NR), 14.01 (NR)  Time x treatment interaction:  post: p= 0.11;  8 weeks: p= 0.44;  1 year: p= 0.95. |  |
| Stanbury 2020 | EMDR (10) vs PE (10) | 6 weeks,  3 months,  6 months | DASS-42 Depression: From pre-therapy to 6 weeks, 3 months, 6 months:  EMDR: 12.6 (15); 10.4 (14.76); 6.4 (16.25)  TF-CBT: 11.5 (10.18); 7.10 (13.51); 6.9 (11.43)  Group x time interaction: F=0.44, non-significant (both groups improved) | DASS-42 Anxiety: From pre-therapy to 6 weeks, 3 months, 6 months  Group x time interaction: F=0.68, non-significant (both groups improved) |
| **Adult, treatment, TF-CBT, early,** | | | | |
| Ironson, 2021 | EMDR (34) vs SMT (37) | 1, 3 and 6 months | BDI-II:  From baseline to 1, 3 and 6 months:  EMDR: 6.96 (NR); 10.39 (NR); 8.93 (NR)  SMT: 5.2 (NR) 7.9 (NR); 7.30 (NR)  Note: mean change from baseline calculated  There was a significant linear group-by-time interaction [F(1,239) = 4.90, p=0.028], but only a trend for a quadratic group-by-time interaction [F(1,239) = 3.30, p =0.070)] such that the EMDR group showed a faster decline in BDI scores than the PFA group, with the SMT group being intermediary. |  |
| **Adult, treatment, wait list/usual care, delayed** | | | | |
| Encinas, 2019 | EMDR-PRECI (14) vs CAU (12) | 90 days (post treatment) | HADS, mean (SD), pre-treatment, post-treatment, 90 days:  EMDR-PRECI: pre-: 6.71 (4.28); 3.85 (2.90) 3.35 (2.89); mean change from baseline (SD): post: 2.86 (4.10); 90 days: 3.36 (4.10)  WL: 6.36 (3.95); 5.90 (3.59); 6.72 (4.64); mean change from baseline (SD): post-: 0.46 (4.14); 90 days: -0.36 (4.74)  Significant effect for time (F (2, 46) = 4.35, p <.05,) and interaction effects between time and group. (F (2, 46) = 4.64, p <.05).  No significant effect for group was founded. For the EMDR group significant differences were found between pre- and post- p<.05). For the WL group, no significant differences were found between means in all comparisons.  Comparison between groups showed significant differences for time 3, p <.05 | HADS, mean (SD), pre-treatment, post-treatment, 90 days:  EMDR-PRECI: 12.28 (2.86); 5.64 (2.87); 5.50 (2.44); mean change from baseline (SD) : post-: 6.64 (3.14); 90 days: 6.78 (2.92)  WL: 10.18 (3.37); 10.81 (4.23); 10.36 (4.92); mean change from baseline (SD) : post-: -0.63 (4.22); 90 days: -0.18 (4.72)  Significant effect for time (F (2, 46) = 8.92, p <.001), significant interaction effect between time and group (F (2, 46) = 11.33, p <.001) and significant effect for  group (F (2, 23) = 7.36, p <.05).  For the EMDR group significant differences were found between pre- and post-,, t (13) = 5.77, p<.001, d=1.63.  No such significant differences were observed in the WL group.  Comparison between groups  showed significant differences comparing post and 90 day  measurements, p<.001, and p< .05, respectively |
| Farrell, 2023 | EMDR-VGTEP (50) vs WL (45) | 6 months | Patient Health Questionnaire (PHQ-9), mean (SD) Note: discrete data NR for subclinical (prevention) and clinical (treatment) PTSD groups; these are overall data.  Both groups: Pre-treatment to 6 months: 11.7 (5.68) to 4.54 (4.94). Change from baseline, mean (SD): 7.25 (5.84). A significant reduction in both groups (EMDR VGTEP vs WL) over time F(5–190) = 43.3, p<0.001 | Generalized Anxiety Disorder Assessment (GAD-7), mean (SD) Note: discrete data NR for subclinical (prevention) and clinical (treatment) PTSD groups; these are overall data.  Both groups: Pre-treatment to 6 months: 11.2 (4.91); 4.73 (4.27). Change from baseline, mean (SD): 6.47 (5.06). A significant reduction in both groups (VGTEP EMDR vs WL) over time F(5–190) = 42.3, p<0.001 |
| Jarero, 2018 | EMDR-IGPT-IOS (35) vs no treatment (30) | 90 days (post treatment) | HADS, mean (SD), pre-treatment, 90 days:  EMDR- IGPT-OTS: 8.94 (3.47); 5.54 (4.22); mean change from baseline (SD) : 3.40 (4.26)  WL: 7.81 (3.60); 10.84(3.72); mean change from baseline (SD): -3.03 (4.01)  Significant effect for group, F(1, 59) = 6.50, p > .05, and a significant effect for the interaction between time and group, F(1, 59)  = 35.79, p < .001, | HADS, mean (SD), pre-treatment, 90 days:  EMDR- IGPT-OTS: 12.74 (3.70); 7.34 (4.39); mean change from baseline (SD) : 5.40 (4.47)  WL: 11.65 (4.33); 12.19 (3.68); mean change from baseline (SD) : -0.54 (4.50)  Significant effect for time (F[1, 59] = 12.86, p <0.001, a significant effect for group (F[1, 59] = 5.56, p < .05), and a significant interaction effect between time and group (F[1, 59] = 19.19, p < .001) |
| Jarero, 2019 | EMDR-PRECI (30) vs no treatment (30) | 90 days (post treatment) | HADS, mean (SD), pre-treatment, 90 days:  EMDR-PRECI: 2.63 (2.18); 1.60 (1.22); mean change from baseline (SD) : 1.03 (2.03)  WL: 3.03 (3.09); 3.10 (2.07); mean change from baseline (SD) : -0.07 (2.95)  Significant effects for the interaction between time and group | HADS, mean (SD), pre-treatment, 90 days:  EMDR-PRECI: 4.53 (2.88); 2.90 (1.90); mean change from baseline (SD) : 1.63 (2.74)  WL: 5.33 (4.18); 5.93 (2.18); mean change from baseline (SD) : -0.60 (3.86)  Significant effect for group F(1, 58) = 10.41, p < .05, and a significant interaction effect between time and group F(1, 58) = 37.40, p < .005 |
| Rousseau 2019 | EMDR (18) vs WL/supportive therapy (18) | Average, 3 months after baseline (both groups) | BDI II: From baseline to 3 months, mean (SD).  EMDR: 13.5 (5.02); 5.75 (4.49), significant improvement, p<0.05; mean change from baseline (SD) : 7.75 (5.23).  WL: 12.17 (7.33); 11.58 (8.37), non- significant improvement; mean change from baseline (SD) : 0.59 (8.64).  Time x treatment interaction:  F = 13.74 and p <0.001, significantly favours EMDR |  |
| Zhao 2023 | EMDR (28) vs WL (29) | 12 weeks (post treatment) | Self-rating Depression Scale (SDS). From baseline to 12 weeks, mean (SD).  EMDR: 75.4 (11.6); 58.7 (14.2), significant improvement, p< 0.001;  mean change from baseline (SD) : 16.7 (14.3).  WL: 75.1 (10.1); 68.1 (9.7), significant improvement, p< 0.001; mean change from baseline (SD) : 7.00 (10.85).  Time x treatment interaction:  F = 12.3 and p=0.001, significantly favours EMDR | Self-rating Anxiety Scale (SAS). From baseline to 12 weeks, mean (SD).  EMDR: 66.8 (14.1); 52.8 (15.8), significant improvement, p< 0.001;  mean change from baseline (SD) : 14.0 (16.44).  WL: 67.9 (10.9); 62.6 (11.4), significant improvement, p=0.011; mean change from baseline (SD) : 5.3 (12.22).  Time x treatment interaction:  F = 9.2 and p=0.004, significantly favours EMDR |
| **Adult, treatment, wait list/usual care, early** | | | | |
| Perez 2020 | EMDR-IGTP (40) vs WL (40) | Post-treatment and 90 days  after WL treatment completion | HADS, mean (SD), pre-treatment, post-treatment, 90 days:  EMDR-IGTP: 12.94 (2.54); 7.31 (3.29); mean change from baseline (SD) : 5.63 (6.79); 90 days: 6.83 (3.24)  WL: 12.71 (2.58); 12.84 (2.43); mean change from baseline (SD) : -0.13 (2.75); 90 days: 6.41 (2.45)  Significant interaction effects between time and group: F (3, 216)=80.82, p<0.001 | HADS, mean (SD), pre-treatment, post-treatment, 90 days:  EMDR-IGTP: 14.45 (2.63); 9.48 (2.48); mean change from baseline (SD) : 4.97 (2.80); 90 days: 6.34 (2.98)  WL: 14.15 (2.68); 14.28 (2.1); mean change from baseline (SD) : -0.13 (2.66); 90 days: 5.49 (2.50)  Significant interaction effects between time and group: F (3, 216)=47.72 p<0.001 |
| Shapiro 2018 | EMDR (13) vs WL (12) | 6 months (post treatment) | Patient Health Questionnaire (PHQ-9), mean (SD), pre-treatment, post-treatment, 6 months  EMDR R-TEP: 14.6 (5.9); 9.8 (7.3); 7.7 (7.4); mean change from baseline to 6 months: 6.90 (7.39)  Significant difference in PHQ-9 scores over time: F(2,18)=11.42; p<0.001.  WL: 7.4 (2.3); 11.8 (6.1); 11.4 (2.5); mean change from baseline to 6 months: -4 (2.63)  There was no statistically significant change over time. |  |
| Ironson, 2021 | EMDR (34) vs PFA (37) | 1, 3 and 6 months | BDI-II: From baseline to 1, 3 and 6 months:  EMDR: 6.96 (NR); 10.39 (NR); 8.93 (NR)  PFA: 3.46 (NR) 5.44 (NR); 5.69 (NR)  Note: mean change from baseline calculated  There was a significant linear group-by-time interaction [F(1,239) = 4.90, p=0.028], but only a trend for a quadratic group-by-time interaction [F(1,239) = 3.30, p =0.070)] such that the EMDR group showed a faster decline in BDI scores than the PFA group, with the SMT group being intermediary. |  |
| **Adult, prevention, wait list/usual care, delayed** | | | | |
| Bates, 2023 | EMDR (online) (12) vs CAU (13) | 6 months | HADS, median (IQR) change from baseline to 6 months  EMDR:−2.00 (-3.00, 1.00);  Usual care: 1.00 (-1.50, 2.00) | HADS, mean (SD) change from baseline to 6 months  EMDR:−0.45 (2.30);  Usual care: −0.83 (4.02) |
| Farrell, 2023 | EMDR-VGTEP (50) vs WL (45) | 6 months | Patient Health Questionnaire (PHQ-9), mean (SD) Note: discrete data NR for subclinical (prevention) and clinical (treatment) PTSD groups; these are overall data.  Both groups: Pre-treatment to 6 months: 11.7 (5.68) to 4.54 (4.94). Change from baseline, mean (SD): 7.25 (5.84). A significant reduction in both groups (EMDR VGTEP vs WL) over time F(5–190) = 43.3, p < 0.001 | Generalized Anxiety Disorder Assessment (GAD-7), mean (SD) Note: discrete data NR for subclinical (prevention) and clinical (treatment) PTSD groups; these are overall data.  Both groups: Pre-treatment to 6 months: 11.2 (4.91); 4.73 (4.27). Change from baseline, mean (SD): 6.47 (5.06). A significant reduction in both groups (EMDR VGTEP vs WL) over time F(5–190) = 42.3, p < 0.001 |

BEP: Brief Eclectic Psychotherapy; CAPS: Clinician Administered PTSD Scale; CAU: Care as usual; CBT: Cognitive Behavioural Therapy; DTS: Davidson Trauma Scale; EMDR: Eye Movement and Desensitisation Reprocessing; IGTP-OTS: Integrative Group Treatment Protocol-Ongoing Traumatic Stress; ImRs: Imagery Rescripting; ITQ: International Trauma Questionnaire; NA: Not applicable; NR: Not reported; PCL-C: PTSD Checklist for DSM-5 (PCL-5) Civilian version; PC: Progressive Counting; PE: Prolonged Exposure; PFA: Psychological First Aid; PRECI: Protocol for Recent Critical Incidents and Ongoing Traumatic Stress PTSD: Post Traumatic Stress Disorder; RCT: Randomised Controlled Trial; R-TEP: Recent Traumatic Episode Protocol; SC: Standard Care; TSI-2: SMT: Stress Management with a Trauma focus; TF-CBT: Trauma-focused CBT; TSI-2: Trauma Symptom Inventory 2; VGTEP: Video-conference Group Traumatic Episode Protocol; WL: Waitlist.

Functioning or health-related quality of life were rarely reported (Table S12): EMDR and TF-CBT were reported as having no significant treatment group differences in health-related quality of life. (24) (17, 25).

**Table S12 Functioning and health-related quality of life**

| **Author, year** | **Interventions (n)** | **Follow-ups** | **Quality of life** | **Functioning or dissociative experience** |
| --- | --- | --- | --- | --- |
| Boterhoven 2020, Assmann 2021 | EMDR (81) vs ImRs (74) | Post-treatment, 8 weeks,  1 year | Not reported | Dissociative Experiences Scale:  Time x treatment interaction, t, df, p:  post: 0.47, 139, 0.64;  8 weeks: 1.72, 139, 0.09;  1 year: -0.38, 128, 0.70 |
| Greenwald, 2021 | Intensive EMDR (24) vs PC (30) | 12 weeks | EUROHIS-QOL: Mean (SD)  At 12 weeks, EMDR group  30.13 (5.57) and PC group (29.21, 4.93) showed no significant difference in reported change post treatment, t(38) = 0.55, p=0.59. | Not reported |
| Santarnecchi 2019 | EMDR (17) vs TF-CBT (14) | EMDR: average 4 weeks;  TF-CBT: average 10 weeks | Not reported | Work and Social Adjustment Scale (WSAS):  EMDR: -3, p<0.01  TF-CBT: -9, p<0.01  Group x time interaction:  F (1,13) 3.36, p=0.039 (significantly favoured TF-CBT)  Time x treatment interaction: Time: F.1;13/ = 16.56, p = 0.003; Treatment: F.1;13/ = 9.44, p = 0.009 (both groups significantly improved from baseline): |
| Farrell, 2023 | EMDR-VGTEP (50) vs WL (45) | 6 months | EQ-5D: Mean (SD) change from baseline. Note: discrete data NR for subclinical (prevention) and clinical (treatment) PTSD groups; these are overall data.  Significant difference between pre- (65.02, 17.99) and 6 months (79.19, 14.84) p<0.001. There was no statistical difference between each of the groups (EMDR VGTEP vs WL). | Not reported |
| Bates, 2023 | EMDR (online) (12) vs CAU (13) | 6 months | EQ-5D-5L and VAS:  Mean (SD) change from baseline:  EQ-5D-5L: EMDR:−0.04 (0.14); Usual care: −0.02 (0.15).  EQ-5D-5L VAS: EMDR: 11.2 (13.10); Usual care: 10.33 (15.33) | Not reported |
| Farrell, 2023 | EMDR-VGTEP (50) vs WL (45) | 6 months | EQ-5D: Mean (SD) change from baseline. Note: discrete data NR for subclinical (prevention) and clinical (treatment) PTSD groups; these are overall data.  Both groups: Significant difference between pre- (65.02, 17.99) and 6 months (79.19, 14.84) p<0.001. There was no statistical difference between each of the groups (EMDR VGTEP vs WL). | Not reported |

BEP: Brief Eclectic Psychotherapy; CAPS: Clinician Administered PTSD Scale; CAU: Care as usual; CBT: Cognitive Behavioural Therapy; df:degrees of freedom; DTS: Davidson Trauma Scale; EMDR: Eye Movement and Desensitisation Reprocessing; IGTP-OTS: Integrative Group Treatment Protocol-Ongoing Traumatic Stress; ImRs: Imagery Rescripting; ITQ: International Trauma Questionnaire; NA: Not applicable; NR: Not reported; PCL-C: PTSD Checklist for DSM-5 (PCL-5) Civilian version; PC: Progressive Counting; PE: Prolonged Exposure; PFA: Psychological First Aid; PRECI: Protocol for Recent Critical Incidents and Ongoing Traumatic Stress PTSD: Post Traumatic Stress Disorder; RCT: Randomised Controlled Trial; R-TEP: Recent Traumatic Episode Protocol; SC: Standard Care; TSI-2: SMT: Stress Management with a Trauma focus; TF-CBT: Trauma-focused CBT; TSI-2: Trauma Symptom Inventory 2; VGTEP: Video-conference Group Traumatic Episode Protocol; WL: Waitlist.

**Cost-effectiveness**

**Table S13 Cost effectiveness data extraction**

| **Paper** | | **Cost-effectiveness of psychological treatments for post-traumatic stress disorder in adults** |
| --- | --- | --- |
| **Study Characteristics** | **Author** | Mavranezouli, I., Megnin-Viggars, O., Grey, N., Bhutani, G., Leach, J., Daly, C., Dias, S., Welton, N., Katona, C., El-Leithy, S., Greenberg, N., Stockton, S., Pilling, S. |
|  | **Year of Publication** | 2020 |
|  | **Journal** | PLoS ONE |
| **Study Design** | **Country** | England |
|  | **Population** | Adults presenting in primary care with clinically important PTSD, with symptoms present more than 3 months after the incident. 39 starting cohort age, percentage of women was 51.6%. |
|  | **Perspective (outcomes)** | Unclear |
|  | **Perspective (costs)** | NHS and personal social services in England |
|  | **Analysis Type** | Modelling, hybrid decision-analytic, decision tree followed by 3 state Markov model |
|  | **Outcome Measure** | Quality adjusted life years |
|  | **Time Horizon** | 3 years (6 months decision tree, 2.5 years in Markov Model) |
|  | **Comparators** | TF-CBT, Non-TF-CBT, Combined somatic/cognitive therapies, SSRIs, Combined TF-CBT/SSRIs, Self-help with support, Self-help without support, Counselling, Psychoeducation, No treatment (reflected in waitlist RCT arms) |
|  | **Cost Type** | Intervention costs, costs of managing side effects from medication, and costs relating to the ‘PTSD’ and ‘no-PTSD’ health states including costs of primary, community and secondary healthcare and PSS costs. |
|  | **Discount Rates** | 3.5% annually |
|  | **Year of valuation** | 2017 |
| **Study Outcomes** | **Sensitivity Analysis** | The deterministic sensitivity analysis showed that results were robust to use of alternative values for the risk of relapse, utility and costs. The probabilistic sensitivity analysis did not affect the position of EMDR which remained the most cost-effective option. |

**Table S14 Cost-effectiveness results**

|  |  | **Study Outcomes** | | | | | | |
| --- | --- | --- | --- | --- | --- | --- | --- | --- |
| **Intervention** | **Intervention Duration** | **QALYs** | **Costs (£)** | **Inc. QALYs†** | **Inc. Costs†** | **ICER†** | **Net monetary benefit‡** | **Probability of Cost-effectiveness§** |
| **Cost-effectiveness of psychological treatments for post-traumatic stress disorder in adults** | | | | | | | | |
| EMDR | 9 hours (6 x 1.5 hours sessions) | 1.8 | 2047 | - | - | - | 33928 | 0.34 |
| Combined somatic/cognitive therapies | 4 hours (4 x 1 hour sessions) | 1.77 | 1963 | 0.03 | 84 | 2800 | 33364 | 0.35 |
| Self-help with support | 180 minutes | 1.75 | 2047 | 0.05 | 0 | Dominated | 32880 | 0.32 |
| Psychoeducation | 3 hours (3 x 1 hour sessions) | 1.74 | 1982 | 0.06 | 65 | 1083.3 | 32754 | 0.42 |
| SSRIs | 4 GP visits 0-3 months + 1 visit at 3-6 months | 1.72 | 2143 | 0.08 | -96 | Dominated | 32316 | 0.37 |
| TF-CBT | 13.5 hours (9 x 1.5 hour individual sessions) | 1.74 | 2854 | 0.06 | -807 | Dominated | 32042 | 0.26 |
| Self-help without support | 40 minutes | 1.71 | 2253 | 0.09 | -206 | Dominated | 31865 | 0.41 |
| non-TF-CBT | 9 hours (9 x 1 hour sessions) | 1.73 | 2670 | 0.07 | -623 | Dominated | 31860 | 0.5 |
| Combined TF-CBT/SSRIs | 13.5 hours (9 x 1.5 hour individual sessions) and 4 GP visits 0-3 months + 1 visit at 3-6 months | 1.73 | 3140 | 0.07 | -1093 | Dominated | 31451 | 0.48 |
| No treatment | N/A | 1.67 | 2488 | 0.13 | -441 | Dominated | 30915 | 0.64 |
| Counselling | 10 hours (10 x 1 hour sessions) | 1.69 | 3043 | 0.11 | -996 | Dominated | 30854 | 1 |
| (TF-CBT) Cohen TF-CBT/CPT | 12 hours (12 x 1 hour sessions) | 2.268 | 5188 | -0.027 | -291 | 10777.8 | 40178 | 0.21 |
| EMDR | 6 hours (8 x 45 minute sessions) | 2.241 | 4897 | - | - | - | 39920 | 0.3 |
| Parent training | 9 hours (12 x 45 minute sessions) | 2.244 | 5099 | -0.003 | -202 | 67333.3 | 39788 | 0.39 |
| (TF-CBT) Group CBT | 11 hours (10 x 1 hour sessions and 1 x 1 hour orientation session) | 2.224 | 4798 | 0.017 | 99 | 5823.5 | 39687 | 0.72 |

†EMDR versus each individual other intervention

‡ At a cost-effectiveness threshold of £20,000

§Estimated in a step wise approach, according to which the most cost-effective intervention is omitted at each step, and the probability of cost-effectiveness of the next most cost-effective intervention amongst the remaining treatment options is re-calculated

**References**

1. National Institute for H, Care E. Post-Traumatic Stress Disorder. London, UK; 2018 2018.

2. Dias S, Sutton AJ, Ades AE, Welton NJ. Evidence Synthesis for Decision Making 2: A Generalized Linear Modeling Framework for Pairwise and Network Meta-analysis of Randomized Controlled Trials. Med Decis Making. 2013;33(5):607-17.

3. Higgins JPT, Altman DG. Assessing risk of bias in included studies. Cochrane handbook for systematic reviews of interventions: Cochrane book series. 2008:187-241.

4. Lunn DJ, Thomas A, Best N, Spiegelhalter D. WinBUGS - A Bayesian modelling framework: Concepts, structure, and extensibility. Statistics and Computing. 2000;10(4):325-37.

5. Sturtz S, Ligges U, Gelman A. R2WinBUGS : A Package for Running WinBUGS from R. J Stat Soft. 2005;12(3).

6. Brooks SP, Gelman A. General Methods for Monitoring Convergence of Iterative Simulations. Journal of Computational and Graphical Statistics. 1998;7(4):434-55.

7. Cohen J. Statistical power analysis for the behavioral sciences: Academic press; 2013 2013.

8. Ren S, Oakley JE, Stevens JW. Incorporating Genuine Prior Information about Between-Study Heterogeneity in Random Effects Pairwise and Network Meta-analyses. Med Decis Making. 2018;38(4):531-42.

9. Jensen JA. An investigation of eye movement desensitization and reprocessing (EMD/R) as a treatment for posttraumatic stress disorder (PTSD) symptoms of Vietnam combat veterans. Behavior Therapy. 1994;25(2):311-25.

10. Moghadam SA, Kazemi R, Taklavi S. Comparing the effectiveness of eye movement desensitization reprocessing and cognitive behavioral therapy in reducing post-traumatic stress disorder. Health Psychology Report. 2020;8(1):31-7.

11. Santarnecchi E, Bossini L, Vatti G, Fagiolini A, La Porta P, Di Lorenzo G, et al. Psychological and Brain Connectivity Changes Following Trauma-Focused CBT and EMDR Treatment in Single-Episode PTSD Patients. Front Psychol. 2019;10:129.

12. Encinas M, Osorio A, Jarero I, Givaudan M. Randomized Controlled Clinical Trial on the Provision of the EMDR-PRECI to Family Caregivers of Patients with Autism Spectrum Disorder. Psychology and Behavioral Science International Journal. 2019;11(1):1-8.

13. Jarero I, Givaudan M, Osorio A. Randomized controlled trial on the provision of the EMDR integrative group treatment protocol adapted for ongoing traumatic stress to female patients with cancer-related posttraumatic stress disorder symptoms. Journal of EMDR Practice and Research. 2018;12(3):94-104.

14. Jarero I, Schnaider S, Givaudan M. Randomized controlled trial: Provision of EMDR Protocol for Recent Critical Incidents and Ongoing Traumatic Stress to first responders. Journal of EMDR Practice and Research. 2019;13(2):100-10.

15. Pérez MC, Estévez ME, Becker Y, Osorio A, Jarero I, Givaudan M. Multisite randomized controlled trial on the provision of the EMDR integrative group treatment protocol for ongoing traumatic stress remote to healthcare professionals working in hospitals during the Covid-19 pandemic. Psychol Behav Sci Int J. 2020;15(555920):10.19080.

16. Shapiro E, Laub B, Rosenblat O. Early EMDR intervention following intense rocket attacks on a town: A randomised clinical trial. Clinical Neuropsychiatry. 2018;15(3):194-205.

17. Farrell D, Moran J, Zat Z, Miller PW, Knibbs L, Papanikolopoulos P, et al. Group early intervention eye movement desensitization and reprocessing therapy as a video-conference psychotherapy with frontline/emergency workers in response to the COVID-19 pandemic in the treatment of post-traumatic stress disorder and moral injury-An. Front Psychol. 2023;14:1129912.

18. Ironson G, Hylton E, Gonzalez B, Small B, Freund B, Gerstein M, et al. Effectiveness of three brief treatments for recent traumatic events in a low-SES community setting. Psychol Trauma. 2021;13(1):123-32.

19. Boterhoven de Haan KL, Lee CW, Fassbinder E, van Es SM, Menninga S, Meewisse ML, et al. Imagery rescripting and eye movement desensitisation and reprocessing as treatment for adults with post-traumatic stress disorder from childhood trauma: randomised clinical trial. Br J Psychiatry. 2020;217(5):609-15.

20. Nijdam MJ, Martens IJM, Reitsma JB, Gersons BPR, Olff M. Neurocognitive functioning over the course of trauma-focused psychotherapy for PTSD: changes in verbal memory and executive functioning. The british journal of clinical psychology. 2018;57(4):436-52.

21. Stanbury TM, Drummond PD, Laugharne J, Kullack C, Lee CW. Comparative efficiency of EMDR and prolonged exposure in treating posttraumatic stress disorder: A randomized trial. Journal of EMDR Practice and Research. 2020;14(1):2-12.

22. Zhao J, Chen DY, Li XB, Xi YJ, Verma S, Zhou FC, et al. EMDR versus waiting list in individuals at clinical high risk for psychosis with post-traumatic stress symptoms: A randomized controlled trial. Schizophr Res. 2023;256:1-7.

23. Rousseau PF, El Khoury-Malhame M, Reynaud E, Boukezzi S, Cancel A, Zendjidjian X, et al. Fear extinction learning improvement in PTSD after EMDR therapy: an fMRI study. Eur J Psychotraumatol. 2019;10(1):1568132.

24. Bates A, Golding H, Rushbrook S, Shapiro E, Pattison N, Baldwin DS, et al. A randomised pilot feasibility study of eye movement desensitisation and reprocessing recent traumatic episode protocol, to improve psychological recovery following intensive care admission for COVID-19. Journal of the Intensive Care Society. 2023;24(3):309-19.

25. Greenwald R, Camden AA, Gamache N, Lasser KA, Chapman R, Rattner B. Intensive trauma-focused therapy with victims of crime. European Journal of Trauma & Dissociation Vol 5(3), 2021, ArtID 100146. 2021;5(3).
